# Monogenic Adult-Onset Inborn Errors of Immunity

**DOI:** 10.3389/fimmu.2021.753978

**Published:** 2021-11-17

**Authors:** Frederik Staels, Tom Collignon, Albrecht Betrains, Margaux Gerbaux, Mathijs Willemsen, Stephanie Humblet-Baron, Adrian Liston, Steven Vanderschueren, Rik Schrijvers

**Affiliations:** ^1^ Department of Microbiology, Immunology and Transplantation, Laboratory of Adaptive Immunology, KU Leuven, Leuven, Belgium; ^2^ Department of Microbiology, Immunology and Transplantation, Allergy and Clinical Immunology Research Group, KU Leuven, Leuven, Belgium; ^3^ Faculty of Medicine, KU Leuven, Leuven, Belgium; ^4^ Department of General Internal Medicine, University Hospitals Leuven, Leuven, Belgium; ^5^ Department of Microbiology, Immunology and Transplantation, Laboratory of Clinical Infectious and Inflammatory Disease, KU Leuven, Leuven, Belgium; ^6^ Vlaams Instituut voor Biotechnologie – Katholieke Universiteit (VIB-KU) Leuven Center for Brain and Disease Research, Leuven, Belgium; ^7^ Laboratory of Lymphocyte Signalling and Development, Babraham Institute, Cambridge, United Kingdom

**Keywords:** primary immunodeficiency, genetics, adult-onset, monogenic, mutation, inborn errors of immunity, autoinflammatory disease, common variable immunodeficiency

## Abstract

Inborn errors of immunity (IEI) are a heterogenous group of disorders driven by genetic defects that functionally impact the development and/or function of the innate and/or adaptive immune system. The majority of these disorders are thought to have polygenic background. However, the use of next-generation sequencing in patients with IEI has led to an increasing identification of monogenic causes, unravelling the exact pathophysiology of the disease and allowing the development of more targeted treatments. Monogenic IEI are not only seen in a pediatric population but also in adulthood, either due to the lack of awareness preventing childhood diagnosis or due to a delayed onset where (epi)genetic or environmental factors can play a role. In this review, we discuss the mechanisms accounting for adult-onset presentations and provide an overview of monogenic causes associated with adult-onset IEI.

## 1 Introduction

Inborn errors of immunity (IEI) are a heterogenous group of disorders in which the development and/or function of the immune system is disturbed (1). They result from inborn errors in genes that functionally impact our innate or adaptive immune system (1, 2). As these disorders are genetically driven, a childhood-onset disease and diagnosis before adulthood is expected. However, some patients are diagnosed in adulthood, either because of a lack of awareness preventing childhood diagnosis or due to delayed onset in adulthood. The distinction between the two can also be blurred, with an incremental escalation of symptoms unmasking the underlying IEI at a later age, with retrospective indications of childhood-onset. The majority of adult-onset IEI patients are deemed to have a polygenic etiology, but an increasing number of monogenic adult-onset causes have been identified during the last decade, facilitated by the increased use of next-generation sequencing (NGS) technology (1). Identified and validated culprit mutations in adults by *in vitro* or *in vivo* molecular assays often reveal low penetrance germline mutations in families with incomplete penetrance or acquired (somatic) mutations. In some cases, a phenotype will only become apparent when a patient encounters specific environmental triggers (such as a pathogen) for which a disrupted response or defense was present from birth (3). In addition, physicians treating adults are generally less aware of monogenic diseases, especially those of the immune system. Moreover, patients with adult-onset IEI are often seen by various medical specialists because of multisystemic manifestations, which are often not recognized as one disease and treated accordingly. In this review, we discuss the mechanisms accounting for adult-onset presentations and provide an overview of monogenic causes associated with adult-onset IEIs.

## 2 Mechanisms of Adult-Onset Presentation in Monogenic IEI

**Figure 1:**
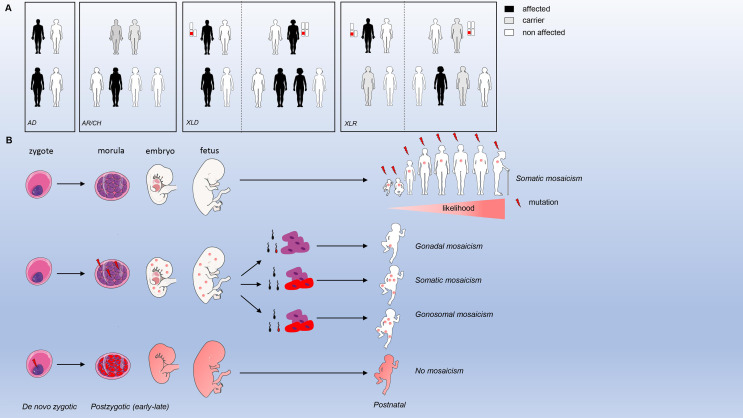
Inheritance modes and mosaicism types. **(A)** modes of inheritance for germline mutations **(B)**. types of mosaicism and origin.

Identification of monogenic defects underlying IEI have increased over time due to the widespread availability of whole exome and genome sequencing. The current international union of immunological societies (IUIS) IEI classification published on January 10, 2020 lists 416 human inborn errors of immunity distributed among 10 groups (1). From those, 64 gene defects (15%) have been discovered (or previously characterized and recently validated) from 2018 till 2020. The majority of these genetic defects have a germline origin (**Figure 1A**), meaning that the mutation is inherited from the father and/or mother. Inheritance can run through an autosomal (AD) or X-linked dominant (one mutation from a (non) affected parent; mother in case of X-linked dominant) or autosomal (AR) or X-linked recessive/compound heterozygous (CH) manner (two identical mutations from each non-affected parent or different mutations in the same gene from each non-affected parent, respectively). In a minority of cases, parents are not (germline) carriers, and a mutation is considered as *de novo* (**Figure 1B**). If a mutation is present in the parental gametes or arises during gametogenesis or conception, then every cell originating from the zygote will have the same mutation. When a mutation arises post-zygotically, 2 or more cell populations with different genotypes co-exist within the same organism (mosaicism). Mosaicism can be further divided into three types a) somatic mosaicism (only affecting somatic cells), b) gonadal mosaicism (only affecting gametes) and c) gonosomal mosaicism (affecting both gametes and somatic cells). Only in the case of gonadal and gonosomal mosaicism, a mutation can be inherited by the offspring.

**Figure 2:**
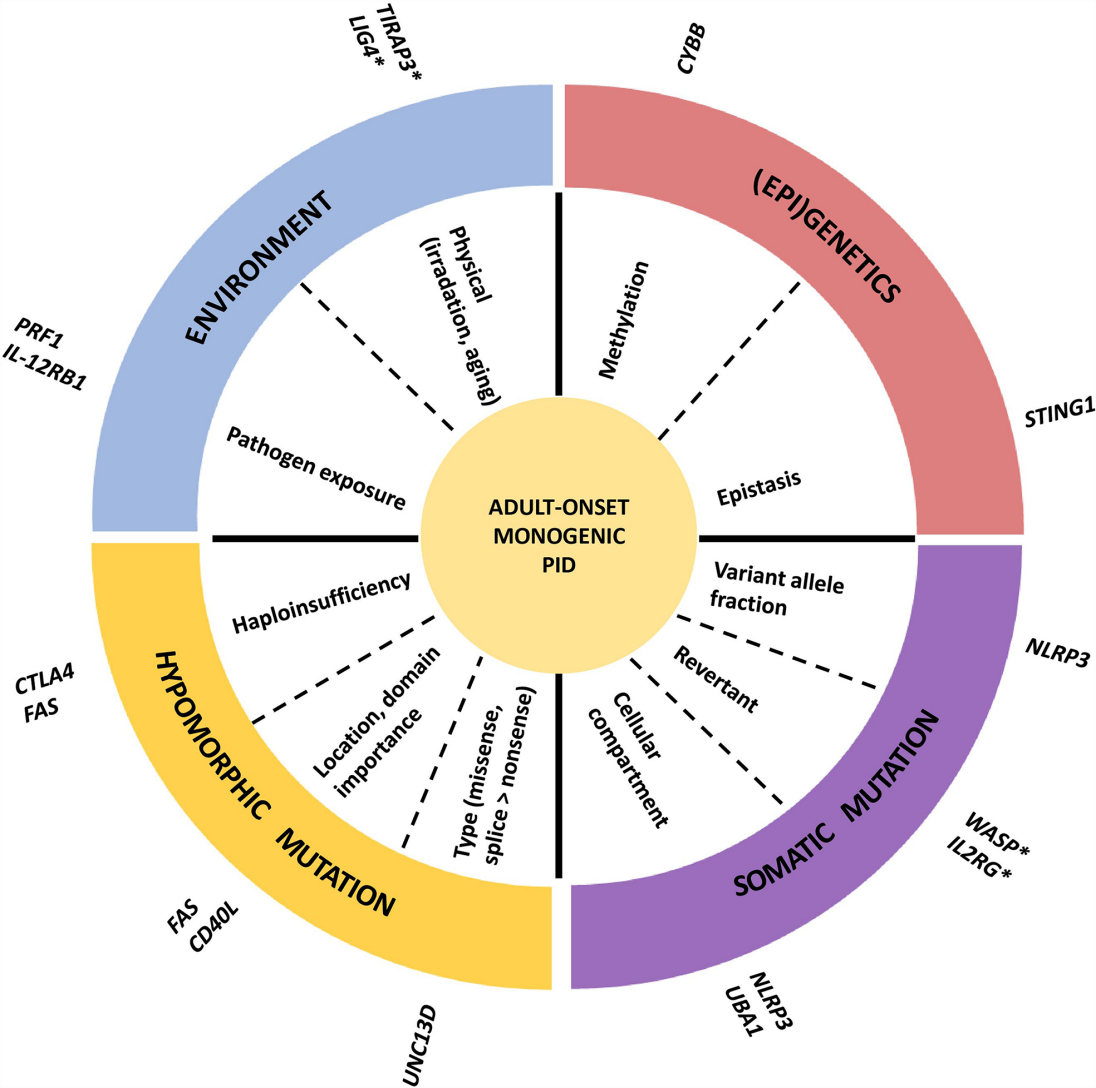
Mechanism of adult-onset IEI. An example of one or two genes is given for every mechanism. *late onset phenotype, but not adult-onset.

The genetic mechanisms contributing to the adult-onset phenotype are summarized in **Figure 2**. Most adult-onset monogenic IEI disease-causing germline mutations are hypomorphic, typically missense mutations (only partially destabilizing functional protein expression) or splice donor/acceptor site mutations (affecting the splicing and processing of mRNA but still allowing for a “leaky” production of the transcript). The impact of the genetic defect is related to the penetrance observed in the affected family, with low penetrance mutations yielding higher chances for adult-onset presentation. For example, in a large Japanese family with X- linked agammaglobulinemia (XLA), a patient (referred to as P2) harboring a splice donor mutation (IVS11+3G>T) in *BTK* resulting in the skipping of exon 11 still had a leaky expression of normal size BTK transcripts resulting in residual BTK protein expression on B cells and peripheral blood mononuclear cells (PBMCs) (4). Another well-described example can be found in Mendelian susceptibility of mycobacterial disease (MSMD), where patients with AR inherited IFNGR1 defects have a complete abrogated signal with an early-onset presentation, while patients with AD inherited defects can remain asymptomatic for a longer time because the mutation still allows for partially retained IFN-γ signaling activity (3). Another example is autoimmune lymphoproliferative syndrome (ALPS-FAS), where homozygous or CH mutations in the *FAS* gene are fully penetrant and present early-onset, while AD mutations are less penetrant with a hierarchy according to the location of the mutation (higher penetrance in the intracellular domain compared to the extracellular domain). However, in some cases, there is no association between the pathogenicity or location of a mutation and the penetrance of a disease. This is demonstrated by IEI in families with *CTLA4* or *NFKB1* haploinsufficiency, where some individuals harbored a complete deleterious variant without a clinical phenotype (5, 6). For *NFKB1*, healthy members carrying the same loss of function (LOF) variant had a similar reduction in protein expression but slight alterations in their immunophenotype (higher CD21 ^low^ B cells compared to affected members), indicating that the cellular penetrance of the same mutation can be different (6). In CTLA4 haploinsufficiency, healthy members with a deleterious mutation had a decreased CTLA4 expression compared to wild-type (WT) healthy controls but increased compared to symptomatically affected family members (5). Therefore, additional disease-modifying factors such as mosaicism, (epi) genetic modifiers, and environmental exposure must play a role in influencing the degree of cellular dysfunction and, thus, the clinical phenotype (7, 8).

Somatic mutations leading to mosaicism are a well-described phenomenon and more common than initially anticipated. A recent study using deep amplicon sequencing found evidence of mosaicism in 30/128 IEI families (23.4%) (9). Mosaicism was most frequently observed in families with *de novo* mutations, families with a moderate-to-high suspicion of mosaicism, and in monogenic autoinflammatory diseases (9). The likelihood of somatic mosaicism increases with age, and this can contribute to an adult-onset disease presentation, either by itself or in cooperation with an inherited germline mutation. Well described examples of IEI with associated somatic mutations can be found in adult patients with autoinflammatory disorders such as cryopyrin-associated periodic syndrome (CAPS due to gain of function (GOF) mutation in *NLRP3*), VEXAS (Vacuoles, E1 enzyme, X linked, Autoinflammatory, Somatic, due to hypomorphic mutations in *UBA1*) syndrome and A20 haploinsufficiency (LOF mutations in *TNFAIP3*). These patients typically have lineage-restricted (mostly myeloid lineage) somatic mutations, indicating that the culprit mutation most likely arose in the later stages of fetal development or during postnatal life (10–14). The threshold minor allelic frequency (MAF) at which a somatic mutation provokes a phenotype is not determined but the progressive increase in MAF over time seems to correlate with disease severity in some patients as described for CAPS (13). This suggests that reduced penetrance in some mosaic IEI is probably a direct consequence of a gene dosage effect. The cellular compartment where a mosaic mutation resides is equally important, as demonstrated by ALPS patients who only harbored a somatic *FAS* LOF mutation in double-negative T cells (not detectable in PBMCs by Sanger sequencing) and demonstrated a phenotype comparable to patients with a germline mutation (15). Besides causing an IEI by itself, a somatic variant can also contribute in addition to a germline variant to a late-onset phenotype, as seen in ALPS-FAS (15). In a case study analyzing 17 individuals with ALPS, 5 of them had presenting symptoms after the age of 16 (16). Two of them had a germline variant with predicted haploinsufficiency in *FAS* and an additional somatic event in the second *FAS* allele which was hypothesized to account for the delayed disease onset. Another IEI where somatic mosaicism is associated with an adult-onset phenotype is a chronic granulomatous disease (CGD). Wolach et al. describe an 80-year-old woman with symptom onset at the age of 66 caused by a stop mutation (p.Tyr30*) in *CYBB* encoding gp91^phox^, important for the oxidative burst response in myeloid cells (17). The mutation was identified in the short-lived blood cells but not in long-lived memory T cells or cheek mucosal cells, suggesting it had occurred later in life. Moreover, leukocytes showed a markedly skewed X inactivation pattern. They hypothesized that this X skewing was caused by another parental gene on the active X chromosome harboring the mutation since no selective advantage has been described for X-CGD mutations in hematopoietic cells. Besides causing disease, somatic mosaicism can also rescue disease, so-called revertant mosaicism. In these cases, a second mutation improves the production of a functional gene product either by giving a selective advantage to WT cells or by directly counteracting the negative impact of the mutant allele on protein function. Although this phenomenon has not yet been reported in adult-onset cases, it is associated with later and milder disease onset as seen in patients with defects in *ADA, IL2RG, WASP, SAMD9* and *CD3-ζ* (18, 19). Finally, epistasis (dependency of the effect of a mutation on the presence or absence of mutations in the same or other genes) can affect disease presentation. In patients with STING associated vasculopathy with onset in infancy (SAVI), caused by GOF mutations in STING, disease-onset and manifestations can be influenced by single nucleotide polymorphisms (SNPs) in STING itself or in other IFN related genes such as *IFIH1* (20).

Environmental factors can also contribute to phenotypic variability, both in terms of severity and age of onset. A typical example is seen in patients who develop disseminated mycobacterial disease after Bacillus Calmette-Guérin (BCG) vaccination and, because of these events, are later diagnosed with an inborn error of IFN-γ immunity. These patients were previously healthy because they were never exposed to this specific microorganism (21). Accordingly, it is hypothesized that delayed contact with specific pathogens in patients with an isolated increased susceptibility for this specific pathogen might account for the delayed onset, as in other cases of MSMD and viscerotropic Yellow fever postvaccination (22). Evidence for infections as a trigger of disease onset also comes from murine models. Knock in mice with a GOF mutation in STING (p.N153S) only developed pulmonary fibrosis after infection with gamma herpes virus 68 (γHV68), and this could be prevented by administration of cidofovir, an antiviral drug against γHV68 (23). It is unclear how these data translate to human patients, but adult-onset pulmonary fibrosis has been reported in SAVI (20). Infections can also trigger immune dysregulation, as observed in patients with familial hemophagocytic lymphohistiocytosis (fHLH), most commonly caused by mutations in *PRF1*, where bouts of autoinflammation often coincide with upper respiratory or gastrointestinal infections (24). Other triggers such as irradiation or chemotherapy can also unmask an underlying DNA repair defect at a later age, as described for patients with Ligase-4 (LIG4) deficiency (25, 26). Age is also an important influencing factor, as humoral immunity can mature and compensate for certain inborn defects in innate immunity. A well-described example is a deficiency in TIRAP3, a key toll-like receptor (TLR) adaptor, caused by recessive LOF mutations. A homozygous LOF mutation was described in a child with severe staphylococcal infection, while older relatives carrying the same mutation were reported to be healthy due to the presence of anti-lipoteichoic acid antibodies targeting *S. aureus*, which were lacking in the index patient (27).

Finally, epigenetic alterations are often implicated to contribute to incomplete penetrance and late-onset disease. Epigenetic modifications play an important role in shaping our immune system and response (28, 29). This is demonstrated by the identification of genetic defects involved in methylation, histone modification, chromatin remodeling and alteration in non-coding RNA resulting in different immunodeficiency syndromes (29). Progressive X-chromosome skewing (XCI) can cause an X-linked recessive IEI in women bearing a pathogenic mutation on one X chromosome. This is demonstrated by cases of CGD due to mutations in *CYBB* in adult women (30, 31). Epigenetic changes are well known to influence the transcriptional expression of genes (29). Therefore, it would not be surprising that the difference in epigenome between two individuals with the same pathogenic mutation can impact the final effect of the mutation in terms of gene expression (32). The impact of epigenetics on disease outcome is best studied in monozygotic twins who are genetically identical. A recent study investigating the epigenome of two monozygotic twins discordant for common variable immunodeficiency (CVID) showed a predominant gain of methylation in critical B lymphocyte genes in the patient compared to his healthy sibling (33). Moreover, the epigenome can change over time, as aging is associated with the relaxation of epigenetic control (34).

## 3 Genetic Defects Associated With Adult-Onset IEI

**Figure 3:**
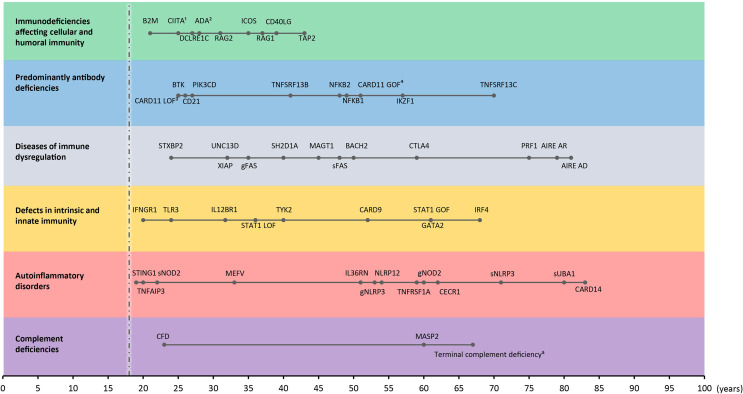
Time of onset in adult IEI. Adult-onset IEI genes with oldest age-of-onset patient reported for every gene (from all reviewed manuscripts) in each category. s: somatic. g: germline. ^1^onset between 20-30 years, ^2^immunological abnormalities, ^3^onset in mid-twenties, ^4^asymptomatic, 51 years old, ^5^genetic defect in C5/6/7/8 or 9, mean age of oldest presentations. Dashed line indicates threshold for adult-onset (18-years-old).

As a guide, we used the most recent IUIS phenotypical classification list (2019) (35) and updates given thereafter (2). We focused on the following groups: immunodeficiencies affecting cellular and humoral immunity, predominant antibody deficiencies, diseases of immune dysregulation, defects in intrinsic and innate immunity, autoinflammatory disorders, and complement deficiencies because adult-onset phenotypes are most frequently reported within one of these seven groups (**Table 1**). Only genes for which there is clearly evidence-based functional studies demonstrating the pathogenic effect of disease-causing mutations *in vitro* or *in vivo* will be discussed. Risk alleles identified by genome wide association studies but lacking a monogenic genotype-phenotype relationship were omitted from this review. Congenital defects of phagocytosis, combined immunodeficiencies with syndromic features, and bone marrow failure syndromes are also not discussed as those rarely present with adult-onset. Appropriate papers were selected from PubMed using the following search terms: “gene name” AND primary immunodeficiency AND adult OR adult-onset OR adult-onset OR incomplete penetrance. Search results were reviewed by two reviewers (FS and TC) and screened for adult-onset patients (no relevant medical history reported before the age of 18, or explicitly reported as adult-onset in the manuscript). Citation history was screened for additional single case reports or series. An age exception was made for the defects affecting cellular and humoral immunity, for which we included late adolescence onset cases with a clear demonstration of hypomorphic variants or cases with very mild symptoms during childhood and diagnosis in adulthood due to worsening symptoms. The oldest patient for each reviewed disease is depicted in **Figure 3**.

**Table 1:** Adult *vs* non-adult-onset associated genes implicated in PID.

Category	Subcategory	Adult-onset	Non-adult-onset
**Immunodeficiencies affecting cellular and humoral immunity**	*T- B+ SCID*	/	CD3D, CD3E, CD3Z, CORO1A, IL2RG, IL7R, JAK3, LAT, PTPRC
	*T- B- SCID*	ADA, DCLRE1C, RAG1, RAG2	AK2, LIG4, NHEJ1, PRKDC, RAC2
	*Combined immunodeficiencies generally less profound than severe combined immunodeficiency*	B2M, CD40LG, CIITA, ICOS, TAP2	BCL10, CD3G, CD40, CD8A, DOCK2, DOCK8, FCHO1, ICOSLG, IKBK, IKZF1, IL21, IL21R, ITK, LCK, LIG4, MALT1, MAP2K14, MSN, POLD1, POLD2, REL, RELA, RELB, RFX5, RFXANK, RHOH, STK4, TAP1, TAPBP, TRFC, TNFRSF4, TRAC, ZAP70
		ADA, DCLRE1C, RAG1, RAG2, CARD11 LOF	
**Predominantly antibody deficiencies**	*Agammaglobulinemia*	BTK, PIK3CD (AD)	BLNK, CD79A, CD79B, IGHM, IGLL1, PIK3R1 (AR), SLC39A7, TCF3, TOP2B
	*CVID*	CD21, IKZF1, NFKB1, NFKB2, TNFRSF13B, TNFRSF13C	ARHGEF1, ATP6AP1, CD19, CD20, CD81, IRF2BP2, MOGS, PIK3R1 (AD), PTEN, RAC2, SEC61A1, SH3KBP1, TNFRSF12, TRNT1
	*Severe reduction in serum IgG and IgA with normal/elevated IgM and normal numbers of B cells, hyper IgM*	/	AICDA (AR), AICDA (AD), INO80, MSH6, UNG
	*Isotype, light chain, or functional deficiencies with generally normal numbers of B cells*	CARD11 (AD GOF)	IGKC
**Diseases of immune dysregulation**	*Familial hemophagocytic lymphohistiocytosis (fHLH syndromes)*	PRF1, STXBP2, STX11*, UNC13D	FAAP24, SLC7A7
	fHLH syndromes with hypopigmentation**	/	APB3B1, AP3D1, LYST, RAB27A
	*Regulatory T cell defects*	BACH2, CTLA4	DEF6, FERMT1, FOXP3, IL2RA, IL2RB, LRBA, STAT3***
	*Autoimmunity with or without lymphoproliferation*	AIRE (AR/AD)	ITCH, JAK1, PEPD, TPP2
	*Immune dysregulation with colitis*	/	IL10, IL10RA, IL10RB, NFAT5, RIPK1, TGFB1
	*Autoimmune lymphoproliferative syndrome (ALPS, Canale Smith syndrome)*	FAS	CASP10, CASP8, FADD, FASL
	*Susceptibility to EBV and lymphoproliferative conditions*	MAGT1, SH2D1A, XIAP	CARMIL2, CD27, CD70, CTPS1, PRKCD, RASGRP1
**Defects in intrinsic and innate immunity**	*Mendelian susceptibility to mycobacterial disease (MSMD)*	IFNGR1, IL12RB1, STAT1 (AD), TYK2, GATA2	CYBB****, IL12B, IL12RB2, IL23R, IRF8 (AD/AR), ISG15, JAK1, RORC, SPPL2A
	*Epidermodysplasia verruciformis (HPV)*	/	CIB1, CXCR4, TMC6, TMC8
	*Predisposition to severe viral infection*	/	FCGR3A, IFIH1, IFNAR1, IFNAR2, IRF7, IRF9, POLR3A, POLR3C, POLR3F, STAT1 (AR), STAT2
	*Herpes simplex encephalitis (HSE)*	TLR3 (AD/AR)	DBR1, IRF3, TBK1, TICAM1, TRAF3, UNC93B1
	*Predisposition to invasive fungal infections*	CARD9	/
	*Predisposition to mucocutaneous candidiasis*	STAT1 (AD)	IL17F, IL17RA, IL17RC, TRAF3IP2
	*TLR signaling pathway deficiency with bacterial susceptibility*	/	IRAK1, IRAK4, MYD88, TIRAP
	*Other inborn errors of immunity related to non-hematopoetic tissues*	/	APOL1, CLCN7, HMOX, NBAS, NCSTN, OSTM1, PLEKHM1, PSEN, PSENEN, RANBP2, RPSA, SNX10, TCIRG1, TNFRSF11A, TNFSF11
	*Other inborn errors of immunity related to leukocytes*	IRF4	IL18BP
**Autoinflammatory disorders**	*Interferonopathies*	CECR1, STING1	ACP5, ADAR1, DNASE1L3, IFIH1, OAS1, RNASEH2A, RNASEH2B, RNASEH2C, SAMHD1, TREX1, USP18
	*Defects affecting the inflammasome*	MEFV (AD/AR), NLRP3, NLRP12	MVK, NLRC4, NLRP1, PLCG2
	*Non-inflammasome related conditions*	CARD14, IL36RN, NOD2, TNFAIP3, TNFRSF1A, UBA1	ADAM17, ALPI, AP1S3, COPA, HAVCR2, IL1RN, LPIN2, OTULIN, PSMB8, PSMG2, PSTPIP1, SH3BP2, SLC29A3, TRIM22
**Complement deficiencies**		C5, C6, C7, C8, C9, *SERPING1*, MASP2, CFD, *CFH, CFI, C3, CD46, THBD, CFHR1-5 (AD/AR), CD59, CFB (AD)*	C1QA, C1QB, C1QC, C1R (AD/AR), C1S (AD/AR), C2, C4A, C4B, CD55, CFB (AR), CFP, FCN3

*STX11: reported in three adult-onsets, but monoallelic and no clear evidence of pathogenicity.

**Adult-onset has been described manifesting as neurological disease but without the presence of immunodeficiency or HLH.

***STAT3: somatic mutations are associated with adult-onset leukemia with autoimmunity and immune-mediated cytopenias.

****CYBB: not reported adult-onset in the context of MSMD, but reported in X-CGD (not in the scope of this review).

Italic: reported in the context of adult-onset aHUS with or without infectious susceptibility or hereditary angioedema.

### 3.1 Immunodeficiencies Affecting Cellular and Humoral Immunity

#### 3.1.1 T- B- Severe Combined Immunodeficiency

##### ADA

Adenosine deaminase (ADA) deficiency is one of the most common forms of severe combined immunodeficiency (SCID) that arises through AR inherited mutations in the *ADA* gene, encoding an enzyme involved in the purine salvage pathway (36). As a form of SCID, ADA-deficiency typically presents at birth, but it has also been described presenting with a milder phenotype later in childhood (delayed onset) or even in adulthood (adult-onset). Late-onset disease tends to gradually worsen over time (36, 50). The first cases that were classified as adult-onset ADA-deficiency were reported by Shovlin et al. (51). They described two sisters that presented in their mid-30s with recurrent infections and chronic respiratory disease. However, retrospectively the older sister developed idiopathic thrombocytic purpura at 17-years-old and the younger sister had a history of recurrent infections, viral warts and mucocutaneous candidiasis since childhood (51). Both had undetectable ADA activity in erythrocytes (lymphocytes not tested) and a CH mutation (p.R211C and a deletion resulting in the loss of exon1). A third 28-year-old male patient was described by Ozsahin et al. (37). He had no relevant medical history apart from recurrent tonsillitis starting at 10-years-old. He carried a CH mutation (p.R101Q acting as a null allele and p.A215T acting as hypomorphic with 15% of WT activity). ADA activity in lymphocytes was very low, but still detectable. The residual ADA activity potentially explains the late onset and mild phenotype in this case (37). In conclusion, although most commonly presenting as SCID during early life, ADA-deficiency is a genetically and clinically heterogenous disorder with different phenotypes depending on the nature and effect of the genetic mutation. (**Table 2**)

**Table 2:** Genes associated with adult-onset combined immunodeficiencies.

	Disease	Genetic defect	Inheritance	Functional defect	Phenotype (key features)	Reference
**DEFECTS AFFECTING CELLULAR AND HUMORAL IMMUNITY**	ADA-deficiency	*ADA*	Germline recessive	LoF mutations, residual enzymatic activity (lymphocytes)	Recurrent and severe infections, low B/T cells	(36, 37)
	Artemis-deficiency	*DCLRE1C*	Germline recessive	LoF, defective dsDNA break repair during VDJ recombination	Susceptibility to viral and fungal infections, predisposition to cancer, myelodysplasia, low B/T cells, neutropenia and thrombocytopenia	(38)
	RAG1-deficiency	*RAG1*	Germline recessive	LoF, reduced VDJ recombination activity	Eosinophilia, chronic dermatitis, low B/T cells	(39)
	RAG-2 deficiency	*RAG2*	Germline recessive	LoF, reduced VDJ recombination activity	Recurrent sinopulmonary infections, CD4 lymphopenia, defective T independent IgG response, hypogammaglobulinemia	(40)
	β2m-deficiency	*B2M*	Germline recessive	LoF, absent or very low β2m surface expression; results in reduced MHC1/FcRn expression	Sinopulmonary infections, cutaneous granulomas	(41–43)
					Hypogammaglobulinemia, hypoalbuminemia, low CD8, normal or elevated IgA and IgM	
	CD40 ligand-deficiency (X-linked hyper IgM syndrome-	*CD40LG*	X-linked	LoF, impaired CD40L-CD40 interaction with decreased B-cell activation and isotype switching	Recurrent infections, low serum IgG, IgA and IgE, normal or increased IgM.	(44)
	MHC II deficiency, complementation group A	*CIITA*	Germline recessive	LoF mutations in CIITA, resulting in absent or decreased MHC II expression.	Recurrent respiratory tract infections, progressive susceptibility to infections, low CD4+ T cells	(45, 46)
	ICOS-deficiency	*ICOS*	Germline recessive	LoF, lack of ICOS expression, severely disturbing T-cell dependent B-cell maturation.	Decreased B-cell numbers, low serum immunoglobulins, recurrent gastrointestinal and sinopulmonary infections, susceptibility to viral infections, autoimmunity and immune dysregulation.	(47, 48)
	MHC-class I deficiency	*TAP2*	Germline recessive	LoF, abrogating TAP2 function/expression, preventing HLA class I molecule maturation.	Granulomatous skin disease, decreased surface expression of HLA class I molecules, (recurrent bacterial infections).	(49)

Additional clinical and immunological features can be found in (35).

##### DCLRE1C

*DLCRE1C* encodes Artemis, a protein involved in the repair of double strand DNA breaks (DSB) induced by recombination-activating gene 1 (RAG1) and RAG2 as a part of VDJ recombination (52). Mutations in *DCLRE1C* result in a form of radiation-sensitive SCID (rs-SCID). Hypomorphic mutations can contribute to later onset disease or leaky SCID, presenting as a combined immunodeficiency at an older age in infancy or childhood (53, 54). One patient reported by Woodbine et al. developed progressive immune dysfunction at 27-years-old (38). She presented with *in situ* carcinoma in one nipple, and progressed to a phenotype with recurrent viral and fungal infections and myelodysplasia. Skin fibroblasts displayed radiation sensitivity and a defective DSB repair in the G2 phase. Genetic analysis revealed a heterozygous mutation (p.P171R) which caused a 3-fold decrease in Artemis activity *in vitro*. The other allele did not show any exonic variants, but the gene product was mis-spliced and prone to nonsense mediated decay, so the authors hypothesized the presence of an intronic splicing mutation. The hypomorphic p.P171R variant in combination with an undefined intronic mutation affecting the splicing of Artemis could contribute to the development of a mild and progressive late onset immunodeficiency (38).

##### RAG1

RAG1 and RAG2 play a crucial role in inducing DSB during VDJ-recombination, and AR null mutations can lead to SCID. Hypomorphic mutations however can give rise to a spectrum of phenotypes including Omenn syndrome, atypical SCID and combined immunodeficiency (55, 56), that can all exhibit features of immune dysregulation (57). Adult-onset phenotypes are very rare, even in patients with hypomorphic mutations in RAG1 who often have mild disease in childhood. Abraham et al. identified a male patient harboring a heterozygous hypomorphic RAG1 frameshift mutation (p.K86Vfs*33) who presented at 38-years-old with a pruritic skin rash that started two years earlier, eosinophilia and T-cell lymphopenia (39). No other mutation in RAG1 or other SCID-related genes that were known at the time were found. The mutation described is damaging as it is found in homozygous or CH SCID patients or patients with Omenn syndrome. His healthy father was found to carry the same mutation, suggesting that other genetic or environmental modifiers might contribute to the observed phenotype (39). Another case reports an adult patient presenting with progressive multifocal leukoencephalopathy at the age of 37 (58). The patient harbored a CH mutation in RAG1 (p.F478Sfs*14 and p.H375D on one allele and a p.R474C on the other allele). A VDJ recombination assay for p.R474C showed 8% of the normal WT function, while the other mutations were not assessed but predicted to be damaging. In retrospect, the patient already was prone to mild infections during childhood (recurrent respiratory tract and gastroenteritis), but infectious manifestations decreased in puberty.

##### RAG2

RAG2 mutations have also been reported to cause an antibody deficiency phenotype (40). A 41-year-old woman with no medical history developed recurrent bronchitis and pneumonia and was diagnosed to have antibody deficiency against bacterial polysaccharide antigens (40). Genetic analysis identified a CH mutation in *RAG2* (p.G95R with absent expression and p.S381fs*1 with a C-terminal truncated RAG2). The functional consequence of the frameshift mutation was not assessed because it was assumed to lead at least to a partial LOF effect based on a mouse model, lacking the C terminal domain of RAG2, which showed partially retained protein expression similar to humans but with impaired lymphoid development (59). This could be a plausible explanation for the adult-onset and mild phenotype in the above described patient.

#### 3.1.2 Combined Immunodeficiencies Less Profound Than SCID

##### B2M

B2M encodes β2-microglobulin (β2m) that composes the light chain of the major histocompatibility complex (MHC) class I molecules and is essential for transport and stabilization of MHC I molecules on the cell surface (41). Furthermore, it also forms a heterodimer with MHC I α chain to form the neonatal Fc receptor (FcRn) which binds IgG and albumin preventing their lysosomal degradation (41, 42). In 1990, two consanguineous siblings were reported suffering from hypoalbuminemia and hypogammaglobulinemia (43). A 34-year-old female patient became symptomatic at 21-years-old, developing a purpuric skin rash and later ulcerative lesions on her leg. She developed thrombocytopenic purpura, and eventually died of sepsis due to bilateral pneumonia. Her 17-year-old brother was asymptomatic, but immunological examination revealed low serum IgG and low albumin levels. Although both siblings were lost to follow up, 35 years later a homozygous LOF *B2M* mutation (p.A11P, leading to <10% expression β2m compared to WT) was found in DNA isolated from preserved sera (42). β2m is also a component of the neonatal Fc receptor (FcRn). FcRn binds IgG and albumin and is important in regulating their normal concentrations (42), thus deficiency of B2M was the culprit for the hypercatabolic hypoproteinemia in these cases. In 2015, two other consanguineous siblings were identified with a homozygous splice site mutation (c.67+1G>T, resulting in nonsense mediated decay and no protein expression in patient’s lymphocytes) (41). They both displayed hypoalbuminemia and low IgG levels, with normal or elevated IgA or IgM levels. The older sister suffered from granulomatous dermatitis since 9 years of age and developed bronchiectasis. Her brother was clinically asymptomatic but shown to have bronchiectasis on pulmonary computed tomography (CT) scan. Based on these two reports, β2m-deficiency can be characterized as an immunodeficiency with hypogammaglobulinemia and hypoalbuminemia that can range from subclinical phenotype to severe symptoms presenting in late childhood or adolescence.

##### CD40LG

*CD40LG* encodes CD40 ligand (CD40L), which is a surface molecule on activated T helper cells that transfers signals through CD40 for B-cell activation, differentiation and isotype switching (60, 61). Mutations in *CD40L* are associated with an X-linked form of hyper IgM syndrome (HIGM) (60), and are the most commonly identified genetic cause of this disorder (44). HIGM syndrome is characterized by low levels of serum IgG, IgA and IgE, normal or high levels of IgM and recurrent infections (44, 60). In a study of 140 patients with HIGM syndrome, 98 patients were found to carry mutations in *CD40LG* (44). Of these, 6 patients had a mild clinical phenotype with onset after 14 years of age. Only one patient had adult-onset disease (23 years) harboring a missense mutation (p.T254M, with proven decreased expression of CD40L) affecting the extracellular domain of CD40L. More recently, a 41-year-old Caucasian man, carrying a nonsense mutation (p.R11*, with normal truncated protein expression) with disease onset in the fifth decade was described by Yong et al. (62). He presented with hemiparesis due to cerebral toxoplasmosis, a 2-year history of recurrent impetigo and chest infections (62) and later progressed to develop eosinophilia and pulmonary aspergilloma (63). The milder phenotype was suggested to be the result of a stable expressed mutant with a normal extracellular domain, still enabling CD40-CD40L interaction and partial signaling. His siblings and other family members were also evaluated in a second study (63). An older brother carried the same mutation. Whereas he did not have any symptoms or relevant clinical history, his IgA and IgG levels were abnormally decreased and his IgM was elevated. In two nephews who suffered from recurrent respiratory infections, the mutation was also found. Their IgG levels, however, were higher than those observed in their two uncles, leading to the hypothesis that the p.R11* mutation might contribute to a gradual deterioration of class switching, or that physiological immune senescence could alter the effects of the mutation (63).

##### CIITA

*CIITA* encodes class II trans-activator, that regulates transcription of MHC molecules by functioning both as a transcriptional activator by coordinating DNA binding factors RFX, CREB and NF-Y and as a transcription factor (64). Deficiency of CIITA results in MHC class II deficiency group A, characterized by a total lack of MHC II expression. In a patient presenting in his twenties, Quan et al. identified a homozygous LOF missense mutation (p.F961S) that resulted in absent expression of MHC II molecules (45). His symptoms progressively worsened in his early thirties, and he eventually died of multiple bacterial infections. Another atypical case was found among three siblings that harbored a homozygous missense mutation (p.L469P), with the mutant allele showing residual activity (46). Two of them suffered from mild immunodeficiency characterized by recurrent respiratory tract infections starting in childhood, while the oldest sister was asymptomatic. Apart from two episodes of pneumonia during childhood, she was completely healthy although she lacked MHC II expression. The investigators hypothesized that the mild clinical phenotype may be due to residual CIITA activity (46).

##### ICOS

Inducible co-stimulator (ICOS) is a surface molecule on activated T-cells and is predominantly expressed on T-cells in the germinal center of secondary lymphoid tissue (47, 65). It plays an important role in induction of several cytokines, including superinduction of IL-10, and T-cell dependent maturation of B-cells (47, 65, 66). Mutations in *ICOS* were first discovered as a genetic cause of CVID by the identification of four adult CVID patients carrying homozygous *ICOS* mutations (c.126-568.del) resulting in a complete lack of ICOS expression (47). A more recent report describing previously published cases expanded the clinical phenotype (48). Six out of 15 ICOS-deficient patients (with age of onset ranging from 1 month to 35 years) presented in adulthood with a variety of symptoms, including recurrent sinopulmonary and gastro-intestinal tract infections, viral infections, signs of autoimmunity and immune dysregulation. They all carried deletions in *ICOS* that resulted in a frameshift and premature stop codon. Opportunistic infections were associated with early disease onset, and were not observed in patients with adult-onset disease (48). In ICOS deficiency, B cell counts appear to decrease during the course of disease (which might explain an adult-onset phenotype), possibly related to progressive bone marrow output failure (48).

##### TAP2

Transporter associated with antigen processing 2 (TAP2) transports antigenic peptides into the ER so that they can be loaded onto human leukocyte antigens (HLA) I molecules (67). *TAP2* is the only gene reported in adult-onset cases of MHC class I deficiency. A homozygous LOF intronic mutation at a splice acceptor site (c.1636-1G>A) introducing a frameshift (p.V545Wfs) was found in a 46-year-old male patient who developed granulomatous skin lesions on his leg and his asymptomatic 30-year-old sister (49). The mutation affected the ATP binding site of TAP2 and abrogated its function, thereby inhibiting the maturation of HLA class I molecules. The index patient had 10-15 times lower expression of HLA class I molecules, but still three times higher than in a cell line derived from a previously described TAP2-deficient patient, possibly contributing to the mild or asymptomatic clinical phenotype in these two siblings (49).

### 3.2 Predominantly Antibody Deficiencies

#### 3.2.1 Agammaglobulinemia

##### BTK

X-linked agammaglobulinemia (XLA) is a humoral immunodeficiency found in males, caused by defects in *BTK* encoding Bruton’s tyrosine kinase, an enzyme involved in B cell maturation by transmitting signals through the pre-B and B cell receptor. Mutations are reported in all domains and result in reduced protein expression or kinase activity. Disease manifestations (recurrent sinopulmonary infections by encapsulated bacteria) mostly occur in infancy (mean age 3.5 years). In exceptional cases, hypomorphic BTK alleles can result in an adult-onset phenotype (**Table 3**) (68, 69, 81). However, when carefully reviewing medical records, most adults diagnosed with XLA will have an early-onset phenotype (70). Among reported adult-onset patients, one patient had symptom onset at 25 years of age (68) and another was diagnosed at the age of 24 when familial work-up, initiated because a sibling was diagnosed with XLA, revealed hypogammaglobulinemia (69). In most reports, late-onset phenotypes are characterized by partial BTK deficiency on flowcytometry and higher immunoglobulin levels compared to early-onset XLA (70, 81) indicative for the hypomorphic impact of the mutation. Noteworthy, B cell counts should always be tested since BTK deficiency can be misdiagnosed for CVID and early-onset GI disease (82)

**Table 3:** Genes associated with adult-onset predominant antibody deficiencies.

	Disease	Genetic defect	Inheritance	Functional defect	Phenotype (key features)	Reference
**PREDOMINANT ANTIBODY DEFICIENCIES**	XLA (X-linked agammaglobulinemia)	*BTK*	Germline X-Linked	LoF, decreased BTK expression resulting in impaired B cel maturation	Sinopulmonary infections by encapsulated bacteria	(68–70)
	APDS1	*PIK3CD*	Germline dominant	GoF, mutations activate class Ia PI3K resulting in PIP3 formation resulting in T cell senescence, mTOR activation, impaired humoral immunity	CVID with EBV susceptibility, autoimmunity and lymphoproliferation	(71, 72)
	CD21 deficiency	*CD21*	Germline recessive	LoF, mutations result in absent CD21 expression, reduced antigen enhancement	Recurrent respiratory tract infections	(73)
	IKAROS haploinsufficiency	*IKZF1*	Germline dominant	LoF, mutations cause haploinsufficiency resulting in B cell maturation defect	CVID, recurrent sinopulmonary infections	(74)
	NFKB1 haploinsufficiency	*NFKB1*	Germline dominant	LoF, mutations cause haploinsufficiency (no expression of mutant allele or dysfunction mutant p105/p50) resulting in impaired canonical NF-kB activation and NLRP3 inflammasome activation	CVID with infectious susceptibility, autoimmunity – autoinflammation, lymphoproliferation and malignancy	(6)
	NFKB2 haploinsufficiency	*NFKB2*	Germline dominant	LoF, mutations cause haploinsufficiency, impaired non-canonical NF-kB activation	Recurrent respiratory tract infections, bronchiectasis. Endocrinopathies, autoimmunity in childhood.	(75)
	TACI deficiency	*TNFRSF13B*	Germline dominant/recessive	LoF, mutations cause haploinsufficiency leading to decreased B cell responsiveness and impaired B cell central tolerance	Variable phenotype of CVID with infectious susceptibility to autoimmunity	(76, 77)
	BAFF-R deficiency	*TNFRSF13C*	Germline recessive	LoF, absent BAFF-R expression	CVID , increased infectious susceptibility	(78)
	CARD11 LOF or GOF (BENTA disease)	*CARD11*	Germline dominant/recessive	LoF, hypomorphic or dominant negative , impaired TCR induced NF-kB activation	LOF: CVID, atopy, cutaneous viral infections, neutropenia	(79, 80)
				GoF, mutations cause spontaneous aggregation of signaling clusters with BCL10, MALT1 and active IKK causing constitutive NF-kB activation	BENTA disease: B cell lymphocytosis, lymphoma and susceptibility to mollusca contagiosum	

Additional clinical and immunological features can be found in (35).

##### PIK3CD

GOF mutations in *PIK3CD* lead to activated phosphoinositide 3-kinase (PI3K) delta syndrome (APDS) type 1. They can be found in patients allegedly categorized as adult-onset CVID with Epstein-Barr virus (EBV) susceptibility, autoimmunity and lymphoproliferation. *PIK3CD* encodes the catalytic subunit (PI3K delta) of a class Ia PI3K, which is expressed in leukocytes and catalyzes the production of the second messenger phosphatidylinositol (3,4,5)-triphosphate (PIP3). GOF mutations in this catalytic subunit result in excessive production of PIP3, leading to activation of the mechanistic target of rapamycin (mTOR) pathway and other kinase complexes such as BTK and inducible T cell kinase (ITK) (83). Subsequently, this results in T cell senescence, lymphoproliferation and impaired antibody responses (83). Adult diagnosed cases were first observed in a large cohort (n=53) of ADPS patients, although adult-onset phenotypes were considered exceptional (71). Five patients (9.4%) were diagnosed in adulthood, one suffered recurrent respiratory tract infections and a local granulomatous skin reaction in response to BCG vaccination in childhood, one was evaluated for chronic cervical lymphadenopathy, two had bronchiectasis and one was asymptomatic. No further details on the disease onset was provided (71). In the European Society for Immunodeficiencies (ESID) APDS registry compiling 51 APDS1 patients, most had disease-onset before the age of 15, but also here adult-onset cases are reported (72). One APDS1 case developed symptoms at the age of 27, although recurrent upper respiratory infections during adolescence might suggest earlier-onset of disease (84).

A second type of APDS (APDS2) is caused by autosomal recessive LOF mutations in *PIK3R1* encoding the regulatory subunit of class Ia PI3K. APDS2 patients present at infancy with severe bacterial infections and autoimmunity, adult-onset cases have not yet been reported.

#### 3.2.2 CVID

##### CD21

CD21 is part of the B cell receptor complex. It recognizes complement component 3d (C3d)-opsonized immune complexes and enhances antigen-specific B cell responses. A compound heterozygous mutation, resulting in complete loss of CD21 surface expression, has been described in one adult with recurrent infections, reduced class-switched memory B cells and hypogammaglobulinemia (73). He had frequent childhood respiratory tract infections, resolving after tonsillectomy at the age of 6 years, followed by an asymptomatic period of 20 years.

##### IKZF1

*IKZF1* encodes IKAROS, a transcription factor belonging to the Ikaros zinc finger transcription factor family, essential for the regulation of lymphocyte differentiation, especially of the B cell lineage (85). The first case of heterozygous mutations in *IKZF1* causing haploinsufficiency and a phenotype reminiscent of CVID was reported in 2016 (74). Eight out of 29 patients had an adult-onset phenotype (19-57 years, all had infections as presenting symptom) and most of them (n=6) were reported within one family with a 4.7 Mb deletion including *IKZF1* on chromosome 7 (in contrast to other families with missense mutations or a very small deletion within the IKZF1 gene). Slowly progressive loss of B cells over time in IKAROS deficiency could provide an explanation for late-onset phenotypes. Aging has been associated with impaired B cell development in the bone marrow, and a mutation in a gene essential for B cell differentiation, such as IKAROS, might accelerate this process (86).

##### NFKB1

NFKB1 is one of the five nuclear factor kappa-light-chain-enhancer of activated B cells (NF-κB) proteins, encoding the p105 subunit. This is post-translationally processed into a p50 subunit, functioning as a transcriptional enhancer of NF-κB target genes when heterodimerized with RelA (p65). NFKB1 haploinsufficiency was first described in 2015 in three families with a CVID phenotype and incomplete penetrance. Within the families, the age of onset was highly variable (2 years to 65 years) with diverse phenotypes ranging from asymptomatic to CVID with malignancy, autoimmunity and bronchiectasis. All reported mutations (splice donor, deletion and frameshift) resulted in rapid degradation of the mutant transcript and residual p105/50 was expressed from the WT allele (87). NFKB1 haploinsufficiency is considered one of the most frequent genetic causes of CVID (6). Incomplete penetrance is prototypical and adult-onset disease is frequently observed (6).

##### NFKB2

Like NFKB1, NFKB2 is one of the five NF-κB proteins, encoding the p100 protein, which is post-translationally processed to a p52 protein. In contrast to p50 which plays a key role in the canonical NF-κB pathway, p52 functions as transcription factor in the non-canonical NF-κB pathway which is important in lymphoid organ development, B and T cell maturation, thymic selection and innate antiviral immunity (75). In a recent review summarizing clinical features on 50 reported patients with NFKB2 haploinsufficiency, 2 were reported as adult-onset (31 and 48 years) and had recurrent respiratory tract infections and bronchiectasis (75).

##### TNFRSF13B

*TNFRSF13B* encodes the transmembrane activator and calcium modulator and cyclophilin ligand interactor (TACI) belonging to the Tumor necrosis factor (TNF) receptor superfamily. This receptor is mainly expressed on B cells and its activation by ligands such as a proliferation-inducing ligand (APRIL) or B-cell activating factor (BAFF) activates a signaling cascade that is involved in B cell differentiation, removal of autoreactive B cells, immunoglobulin production and class switching. Both heterozygous and homozygous LOF mutations have been linked to adult-onset CVID and can be found in 5-10% of CVID patients (76). Heterozygous mutations are associated with autoimmunity and lymphoproliferation, more than biallelic mutations, possibly because B cells are still partially responsive to allow for autoimmune complications (77). Its role as a monogenic cause of IEI has been debated and a role as a modifier gene in CVID postulated (88).

##### TNFRSF13C

*TNFRSF13C* encodes the BAFF factor receptor (BAFF-R). Similar to TACI it belongs to the TNF receptor superfamily, and is mainly expressed on B-cells. Upon binding of its ligand, downstream pathways are activated that regulate B cell survival and maturation. BAFF-R deficiency has been described in two adult-onset (37 and 70 years) CVID patients carrying a homozygous LOF deletion in *TNFRSF13C* resulting in absent expression (78). They presented with respiratory tract infections associated with profound B cell lymphopenia and low number of switched memory B cells.

##### CARD11

CARD11 is a scaffold protein that plays a role in TCR and BCR signaling by linking antigen recognition to NF-κB in lymphocytes (79, 89). Mutations in CARD11 can lead to a variety of clinical phenotypes depending on the nature of the mutation (LOF or GOF). In case of LOF, biallelic null mutations result in combined immunodeficiency in childhood. Hypomorphic variants or dominant negative mutations are linked to a milder phenotype that predisposes to a variable immunodeficiency and atopy (79, 89, 90). In a study containing 48 patients with dominant negative CARD11 mutations, three of them were reported with disease onset at 18 years or older (79). One woman had onset at 20 years of age and suffered from neutropenia, hypogammaglobulinemia and indolent LGL. Another patient was 18 years old and apart from atopic disease, she also suffered from bronchiectasis and mollusca contagiosum. Lastly, a female carrying a compound heterozygous mutation was identified with disease onset in her mid-twenties when she developed recurrent bacterial sinopulmonary tract infections and hypogammaglobulinemia. The mutations (p.R47H and p.R187P) carried by the first two patients were shown to exert a dominant negative effect by interfering with WT CARD11, resulting in decreased NF-κB signaling. The last patient carried a compound heterozygous LOF mutation (p.R912Q and p.D1152N). In case of a germline GOF mutation, a B cell lymphoproliferative disorder known as B cell Expansion with NF-κB and T cell Anergy (BENTA) can occur. Patients with BENTA often present with susceptibility to molluscum contagiosum and polyclonal B cell lymphocytosis (91). Although often seen shortly after birth or in childhood, a clinically asymptomatic adult individual has been reported (80).

### 3.3 Diseases of Immune Dysregulation

#### 3.3.1 Familial Hemophagocytic Lymphohistiocytosis

Familial hemophagocytic lymphohistiocytosis (fHLH) represents a group of immune dysregulation disorders associated with uncontrolled activation of histiocytes and T cells. The pathogenesis of fHLH centers around the impaired cytolytic function of natural killer (NK) cells and cytotoxic T cells, which hinders the clearance of antigens and eventually results in a hyperinflammatory state (i.e. cytokine storm) (118). Classical symptoms include cytopenia, prolonged fevers, hepatosplenomegaly, hypertriglyceridemia, hyperferritinemia and neurological disease. The diagnosis of fHLH can be established using the HLH-2004 criteria (119). Several defects in genes important for cytolytic function have been validated as cause of fHLH and some of them (described below) were unmasked after a first episode in adulthood (**Table 4**).

**Table 4:** Genes associated with adult-onset diseases of immune dysregulation.

	Disease	Genetic defect	Inheritance	Functional defect	Phenotype (key features)	Reference
**DISEASES OF IMMUNE DYSREGULATION**	FLH2 (Familial Hemophagocytic Lymphohistiocytosis 2)	*PRF1*	Germline recessive	LoF, lower/absent expression of perforin leading to defective perforin-dependent cytotoxic pathway and decreased NK and CTL function.	HLH	(92–94)
	FLH3 (Familial Hemophagocytic Lymphohistiocytosis 3)	*UNC13D*	Germline recessive	LoF, defective cytotoxic granule exocytosis, leading to decreased NK and CTL function.	HLH	(94–96)
	FHL5 (Familial Hemophagocytic Lymphohistiocytosis 5)	*STXBP2*	Germline recessive	LoF, defective granule exocytosis in NK and CTL.	HLH	(94, 97)
	BRIDA (BACH2-related immunodeficiency and autoimmunity)	*BACH2*	Germline dominant	LoF, haploinsufficiency	CVID, colitis, recurrent respiratory tract infections.	(98)
	CTLA-4 haploinsufficiency	*CTLA-4*	Germline dominant	LoF, haploinsufficiency	Hypogammaglobulinemia, susceptibility to infections, autoimmune manifestations.	(5, 99)
	APECED (Autoimmune Polyendocrinopathy Candidiasis Ectodermal dystrophy)	*AIRE*	Germline dominant/recessive	LoF, dominant negative mutations in PHD1 domain, causing mutant protein to form non-functional homo-oligomers by associating with WT AIRE. LoF, recessive,	Mucocutaneous candidiasis, hypoparathyroidism, adrenocortical insufficiency, isolated organ-specific autoimmunity.	(100–103)
	ALPS-FAS (Autoimmune Lymphoproliferative syndrome)	*FAS*	Germline dominant/somatic	LoF, ICD mutations: dominant negative effect on WT protein. ECD mutations: haploinsufficiency. Disturbed lymphocyte homeostasis through defective apoptosis.	Non-malignant lymphoproliferation, lymphadenopathies, splenomegaly, increased risk for lymphoma, increased DNT, cytopenias	(104–107)
	XMEN (X-linked immunodeficiency with magnesium defect, Epstein-Barr virus infection, and neoplasia)	*MAGT1*	X-linked	LoF, disturbed N-linked glycosylation, affecting function of MAGT1-dependent immunoglycoproteins.	Immune dysregulation, chronic EBV infection, EBV-related lymphoproliferation, autoimmune cytopenias, magnesium defect, splenomegaly, liver abnormalities, intellectual disability.	(108–110)
	X-linked lymphoproliferative syndrome 1 (XLP-1)	*SH2D1A*	X-linked	LoF, resulting in disturbed SAP-mediated signal transduction.	Susceptibility to EBV infections, HLH, dysgammaglobulinemia, lymphoma.	(111–114)
	X-linked lymphoproliferative syndrome 2 (XLP-2)	*XIAP*	X-linked	LoF, resulting in increased sensitivity to apoptosis and disturbing XIAP-mediated signaling.	Susceptibility to EBV infections, HLH, hypogammaglobulinemia, lymphoma, IBD, splenomegaly.	(115–117)

Additional clinical and immunological features can be found in (35).

##### PRF1

*PRF1* encodes perforin, highly expressed by cytotoxic T and NK cells. Perforin is a pore forming protein involved in cell death. A case study reports on an adult Japanese patient presenting with a first episode of HLH at the age of 62 years (92). Genetic analysis on PBMCs and nails revealed a germline compound heterozygous *PRF1* mutation (p.L364fs and p.V306I). The compound heterozygous mutation was hypomorphic as observed by the decreased but not abolished perforin expression *in vitro*. Another report from 2002 describes 2 siblings (brother and sister) who presented at the age of 21 and 22, respectively, with a first episode of HLH (93). HLH was preceded by a respiratory tract infection in one sibling and a diagnosis of T cell lymphoblastic lymphoma in the other sibling. Both of them carried a compound heterozygous mutation resulting in a missense (p.A91V) and stop mutation (p.W374*) in *PRF1* with decreased perforin expression. Lastly, one of the largest cohorts to date studying 175 adult patients (out of a total of 1531 patients who were referred for suspected HLH), observed genetic defects in *PRF1* in 18 of them (age 18-75 years) (94). Of the patients with available perforin expression, only 1 had absent perforin expression, while 6 had low expression and 2 had normal expression (in the presence of an *in silico* predicted pathogenic heterozygous mutation). In conclusion, adult-onset presentation is associated with hypomorphic *PRF1* variants leading to decreased but not abolished perforin activity in most patients and/or requires an additional trigger such as infections or malignancy, similar to secondary HLH.

##### STXBP2

*STXBP2* encodes syntaxin-binding protein 2, which plays a role in the regulation of intracellular granule trafficking in neutrophils, NK cells and mast cells. Almost all reported patients presented during the neonatal period or in early infancy. In a case series by Meeths et al. reporting on 8 patients, the oldest patient presented at the age of 17 and HLH was preceded by an EBV infection (97). She carried compound heterozygous mutations in *STXBP2* resulting in a missense (p.S545L) and stop (p.Q432*). NK cell cytolytic activity and degranulation were both impaired. In the cohort described by Zhang et al., one patient had disease onset at 24 years of age. He was found to carry a homozygous mutation (c.1782*12G>A) in the 3’UTR of the *STXBP2* gene (94). He had significant decreased NK cell cytotoxic function and perforin activity suggesting a partial defect. Although very rarely reported in general in association with fHLH, mutations in *STXBP2* should be considered when assessing adult patients with HLH.

##### UNC13D

*UNC13D* encodes the Munc13-4 protein which has a priming function for cytotoxic granules secretion before fusing to the vesicle membrane (95). The first report described patients from 7 families with infancy or adolescence onset of fHLH (1.5 months – 13 years) caused by homozygous or compound heterozygous LOF (frameshift, stop or deletions) mutations in *UNC13D*. These mutations resulted in defective release of lytic enzymes from T cell receptor activated lymphocytes (95). Rohr et al. described 1 patient with a first HLH episode at 34 years carrying a compound heterozygous mutation (missense and frameshift leading to a premature stop) (96). Zhang et al. identified 7 adult-onset fHLH (18 – 30 years) associated with eight *UNC13D* variants (5 missense, 2 splice site and 1 intronic) not reported in healthy controls either in heterozygous or compound heterozygous state (94). Two out of three patients (both with a heterozygous c.753 +3G>A splice site mutation) tested for NK cell function *in vitro* had absent cytotoxic activity. However, for some mutations, *in silico* scores predicted a benign effect of the mutation (p.E725G, p.S747N) or mutations were located in non-conserved regions (p.R527W, p.S747N), despite being absent in healthy controls. Therefore, given the lack of functional validation, it remains unclear whether all of these mutations are causative for fHLH.

#### 3.3.2 Regulatory T Cell Defects

##### BACH2

BACH2 is a transcription factor in T- and B-lymphocytes that regulates differentiation and maturation, and is important in the suppression of inflammation (98, 120).

BACH2-related immunodeficiency with autoimmunity (BRIDA) is a recently discovered disorder caused by LOF mutations in the *BACH2*, resulting in haploinsufficiency (98). Afzali et al. (98) discovered two LOF mutations that have been predicted to disrupt protein stability and prevent dimerization (p.L28K) or interfere with protein localization in the nucleus by aggregation in the cytoplasm (p.E788K). All patients suffered from inflammatory bowel disease-like symptoms and recurrent respiratory tract infections which was attributed to CVID. In two out of three patients (one carrying the p.L28K-mutation and the other carrying the p.E778K-mutation) disease onset was in childhood, while the third patient (harboring the p.E788K-mutation) developed symptoms in the sixth decade. This suggests a variability in clinical phenotype, with the possibility of adult-onset disease (98).

##### CTLA4

CTLA4 is a surface molecule expressed on T cells, which binds to B7 molecules on antigen presenting cells (APC), thereby competing with the co-stimulatory molecule CD28. As a result, suppressive functions of regulatory T cells (Tregs) are stimulated and proliferation of effector T cells is inhibited (99). CTLA4 haploinsufficiency, caused by LOF mutations in *CTLA4*, was first described in 2014 in families with AD inherited form of immune dysregulation, characterized by hypogammaglobulinemia, infectious susceptibility and auto-immunity (99, 121). As for most other monogenic diseases caused by haploinsufficiency, penetrance was incomplete and adult-onset symptoms (up to 40 years of age at disease onset) were common (8). Functionally CTLA4 haploinsufficiency led to impaired transendocytosis and suppressive activity of Treg cells, explaining the susceptibility to autoimmunity (99). The largest cohort to date of CTLA4 haploinsufficiency describes 133 patients from 54 different families, of whom 12 (9.0%) had symptoms after the age of 18 years (18-59 years) (5). Both unaffected and affected members had a similar cellular penetrance *in vitro*, suggesting that additional factors (hitherto not identified) influence the clinical phenotype and disease onset.

#### 3.3.3 Autoimmunity With or Without Lymphoproliferation

##### AIRE

Autoimmune polyglandular syndrome type 1 (APS-1) is a disease characterized by a classic triad of chronic mucocutaneous candidiasis (CMC), hypothyroidism and adrenocortical insufficiency (two of three criteria need to be present for a clinical diagnosis). APS-1 is caused by autosomal recessive mutations in *AIRE*, encoding a protein expressed in thymic medullary epithelial cells which mediates the ectopic expression of tissue restricted proteins to developing T cells. It is essential to regulate self-tolerance and promotes the negative selection of autoreactive T cells, which can cause autoimmunity (122). AIRE deficiency patients reported in large cohorts from different countries (100, 101, 123) often have infancy onset symptoms, but are mostly diagnosed in later life when cumulative autoimmune phenomena alert physicians for monogenic causes. Autoimmune manifestations in adulthood in the context of recessive mutations is very rare and careful history for milder manifestations in infancy is important. In contrast to recessive mutations, dominant negative mutations in *AIRE* are commonly associated with organ specific autoimmunity (pernicious anemia, vitiligo, autoimmune thyroiditis) presenting in adulthood (for adults range 21-81 years in Oftedal et al.) (102, 103). Some of the mutation carriers are even asymptomatic with or without the presence of autoantibodies (102). Dominant negative mutations are clustered within the plant homeodomain of the AIRE protein, preventing the binding of AIRE to histone H3, thereby negatively impacting its transcription and transactivation activity. The dominant negative effect can be explained by the fact that AIRE functions as a homotetramer. Incomplete penetrance occurs because the formation of WT AIRE tetramer is still possible and can be sufficient to induce self-tolerance in some individuals. In addition, the strength of the dominant negative effect also correlates with the location of the mutation in the PHD domain, accounting for the phenotypical diversity (102).

#### 3.3.4 Autoimmune Lymphoproliferative Syndrome

ALPS is a disease characterized by benign lymphoproliferation, autoimmunity (mostly cytopenias), susceptibility to lymphomas and a high proportion of double negative T cells (DNT) due to a defective lymphocyte homeostasis by inherited defects in apoptosis genes (104). Clinical diagnosis is guided by available criteria (124) and further classification in subtypes is based on genetic analysis (gene and mode of inheritance): ALPS FAS (*FAS* homozygous or heterozygous, germline), ALPS-sFAS (*FAS*, somatic), ALPS-FASLG (*FASLG*, germline), ALPS-CASP10 (*CASP10*, germline) or ALPS-U (unknown genetic defect but meets diagnostic criteria) (124). To our knowledge, only ALPS-FAS, ALPS-sFAS have been observed in adult-onset presentations.

##### FAS

The majority of ALPS patients have a genetic defect in *FAS*, encoding a member of the TNF-superfamily, which is expressed as a homotrimer on B and T cells and essential in the regulation of apoptosis (124). In contrast to recessive mutations in FAS, only found in a minority of ALPS patients, heterozygous germline mutations either leading to a mutant FAS protein (with dominant negative action) or decreased FAS protein expression (haploinsufficiency) are more frequently encountered and associate with an early-onset disease (infancy life) but incomplete penetrance (<60%) (105). The penetrance correlates with the location of the mutation in the protein structure (lower in the extracellular compared to intracellular domain, because mutations in the extracellular domain mostly led to haploinsufficiency) (105). In a cohort of 90 patients with ALPS-(s)FAS, 7% of affected patients had adult-onset disease (range 18-35 years) with a milder form of lymphoproliferation (106). These patients had more combined germline and somatic mutations or germline mutations in the extracellular domain compared to patients with early-onset disease. The somatic mutation was either acquired on the second *FAS* allele or occurred through somatic uniparental disomy (a situation where both chromosomes are derived from 1 parent). Somatic mutations detected in *FAS* in DNT cells (either dominant negative or haploinsufficiency) were also described in other reports of ALPS-sFAS in adults (43 and 48 years at disease onset) (15, 107).

#### 3.3.5 Susceptibility to EBV and Lymphoproliferative Conditions

##### MAGT1

*MAGT1* is located on the X-chromosome and encodes the magnesium transporter 1, that has a dual function as a plasma membrane magnesium transporter and as a subunit of the endoplasmic reticulum localized oligosaccharyltransferase complex (125). Hemizygous LOF mutations can result in a XMEN syndrome (X-linked immunodeficiency with magnesium defect, Epstein-Barr virus infection, and neoplasia) in males, whereas female carriers are unaffected. The pathogenesis is most likely related to a defective N-glycosylation of key T and NK cell receptors, impairing their function or leading to rapid degradation (108, 109), although defective magnesium influx leading to impaired downstream signaling upon TCR stimulation was also observed in XMEN patients (110). In the first report, adult-onset (45 years) was described in one patient with EBV driven lymphoma (110). Other adult-onset lymphomas have been reported in later reports (109).

##### SH2D1A

X-linked lymphoproliferative syndrome 1 (XLP-1) is a clinically heterogeneous disorder caused by hemizygous mutations in the *SH2D1A* gene, that is characterized by extreme susceptibility to EBV infection, HLH, dysgammaglobulinemia and malignant lymphoma (111, 126, 127). *SH2D1A* encodes SAP (signaling lymphocyte activation molecule (SLAM)-associated protein), which is a cytosolic protein that plays an indispensable role in the signal transduction of T cells, NK cells and Natural killer T lymphocytes (NKT) cells (112, 128). The clinical phenotype is very variable, and although pediatric onset is most common, adult-onset with lymphoma or HLH as primary manifestation has been described (111, 113, 114, 129). In a large cohort study of Booth et al. (111), onset of disease in EBV+ patients ranged from 8 months to 40 years (median 4 years), and in EBV- patients from birth to 31 years (median 3.5 years). So even in the absence of EBV infection, disease was observed in adulthood, meaning that other factors than EBV can influence disease onset. Similarly, a recent case report of a 21-year-old male presenting with EBV+ HLH showed presence of a pathogenic variant in *SH2D1A* (p.E17K), leading to a normally expressed mutant protein with diminished binding to phosphorylated 2B4 receptor (important for NK-cell activation) (114). His siblings carrying the same mutation were unaffected, even after encountering EBV infection. Again, EBV was not a determinant for disease onset. Another report supporting role for genetic confounders comes from Liang et al., describing a 44-year-old female patient presenting with HLH and NK cell leukemia. She harbored a mutation (p.A3S) in the SH domain of SAP, resulting in absent protein expression (129). Targeted sequencing revealed a 28.7% mutant/WT ratio suggestive for a somatic mutation, although this was not confirmed by investigating the presence of the mutation in non-hematopoietic tissue. Somatic mosaicism in combination with X linked skewing (XCI) could explain why this patient had an adult-onset phenotype, but this was not investigated (129).

##### XIAP

Another less frequent cause of an XLP (XLP-2) is caused by deficiency of the X-linked inhibitor of apoptosis (XIAP) (115). XIAP is an important inhibitor of apoptosis that is expressed in different hematopoietic cells such as lymphocytes, myeloid cells and NK cells. Cells from XIAP-deficient patients are shown to have increased sensitivity to apoptosis and patients display almost absent numbers of NKT cells (115). Phenotypically it highly resembles XLP1, with the addition that splenomegaly is often the first presenting manifestation (115, 130) and that XIAP deficiency is associated with inflammatory bowel disease (130). Rigaud et al. characterized a potential modifier gene that contributes to development of disease phenotype in patients harboring a hypomorphic mutation in *XIAP* (p.G466*) (116). In their study, patients carrying a co-segregated CD40-ligand (*CD40L*) polymorphism (p.G219R) in addition to the XIAP defect developed clinical disease manifestations, whereas patients harboring the hypomorphic XIAP mutation alone remained asymptomatic (116). The CD40L mutation was demonstrated to affect B cell differentiation and class switch recombination. Although non-random X-inactivation favoring the WT allele has been described (115), symptomatic female carriers have been identified (117). Aguilar et al. (117) described two heterozygous female patients carrying hypomorphic mutations in XIAP (p.H220Y and p.G466*) that developed inflammatory bowel disease at an adult age (32 years and 28 years). Both displayed a predominant expression of the mutant allele, suggesting that the hypomorphic nature of their XIAP mutations could have contributed to skewed X-inactivation towards the mutant or that the presence of a second undefined mutation gave a selection advantage (117).

### 3.4 Defects in Intrinsic and Innate Immunity

Defects in intrinsic and innate immunity are categorized according to the specific pathogen susceptibility caused by genetic defects. Some of them are rarely or never seen in adults and therefore left out of the scope of this review (ND). Overall, 9 categories exist: a) mendelian susceptibility to mycobacterial disease (MSMD), b) epidermodysplasia verruciformis (ND), c) predisposition to severe viral infection (ND), d) herpes simplex encephalitis (HSE), e) invasive fungal infections, f) chronic mucocutaneous candidiasis, g) TLR signaling pathway deficiency with bacterial susceptibility, h) other inborn errors of immunity related to non-hematopoietic tissues and i) other inborn errors of immunity related to leukocytes (**Table 5**).

**Table 5:** Genes associated with adult-onset defects in intrinsic and innate immunity.

	Disease	Genetic defect	Inheritance	Functional defect	Phenotype (key features)	Reference
**DEFECTS IN INTRINSIC AND INNATE IMMUNITY**	IFNGR1 partial deficiency	*IFNGR1*	Germline recessive	LoF, recessive hypomorphic variants, residual expression on cell surface with impaired response to IFN-γ	MSMD	(3, 131)
			Germline dominant	LoF, dominant negative mutations result in accumulation of non-functional truncated IFNGR1 impeding the normal function of IFNGR1 dimers		
	IL-12RB1 deficiency	*IL12RB1*	Germline recessive	LoF, no expression of IL12RB1 and impaired IL-12/IL-23 signaling	MSMD	(132)
	STAT1 AD deficiency	*STAT1*	Germline dominant	LoF, complete or hypomorphic depending on the location and mechanism (impaired DNA binding of STAT1, impaired phosphorylation or both).	MSMD	(133)
	TYK2 P1140A	*TYK2*	Germline recessive	LoF, lacks catalytic activity leading to impaired IL-23 signaling	MSMD	(134, 135)
	GATA2 deficiency	*GATA2*	Germline dominant	LoF, complex mechanism either leading to haploinsufficiency or mutation induced ectopic activities	Immunodeficiency (viral, fungal, MSMD infections), hematopoietic disorders	(136)
	CARD9 deficiency	*CARD9*	Germline recessive	LoF, impaired cytokine production in response to fungal ligands, neutrophilic killing and Th17 immunity	Invasive fungal infections, CMC	(137)
	STAT1 GOF	*STAT1*	Germline dominant	GoF, impacting STAT1 levels and phosphorylation status	CMC, viral and bacterial infections, invasive fungal infections, autoimmunity, humoral immunodeficiency	(138)
	TLR3 deficiency	*TLR3*	Germline recessive or dominant	LoF, haploinsufficiency, hypomorphic or dominant negative, loss of expression or impaired signaling upon dsRNA stimulation	HSE	(139)
	IRF4 deficiency	*IRF4*	Germline dominant	LoF, decreased DNA binding and ISRE induced transcription	Whipple disease	(140)

Additional clinical and immunological features can be found in (35).

#### 3.4.1 Mendelian Susceptibility to Mycobacterial Disease

MSMD is characterized by predisposition to mycobacterial disease caused by weakly virulent mycobacteria in otherwise healthy individuals. Till present, all genes implied in MSMD play a direct or indirect role in the IFN-γ dependent immunity, crucial to mycobacterial defense.

##### IFNGR1

Mutations in the IFN-γ receptor, composed of a heterodimer of IFNGR1 and IFNGR2, were the first described genetic defects in MSMD (3). Recessive mutations causing a complete deficiency without residual expression cause a severe infancy onset phenotype with life threatening mycobacterial (or some other intracellular bacteria such as Listeria and Salmonella spp.) and viral infections. Hypomorphic recessive LOF mutations in *IFNGR1* (although rarely seen in adulthood) or AD LOF mutations in *IFNGR1* causing a partial deficiency are associated with a milder phenotype and can be observed in later life (3, 131).

##### IL12RB1

IL12RB1 is both part of the IL12R (in combination with IL12RB2) and of the IL23R (in combination with the IL23R), mediating IL-12 and IL-23 signaling. IL12RB1 deficiency, caused by recessive LOF mutations, is the most common genetic defect in MSMD. One of the largest cohorts studying 141 IL12RB1 deficient patients, observed an age of onset between 1 week to 31.7 years (mean age, 2.4 years, SD ± 4.9 years, range 2 weeks to 31.7 years) (132). Most of the cases were caused by BCG vaccination. Eight of the patients remained asymptomatic at the time of publication, even though the *in vitro* cellular penetrance was complete. Together this indicates that exposure to a specific pathogen most likely drives the age of onset in this immunodeficiency.

##### STAT1

STAT1 is an important transcriptional activator mediating cellular responses to pathogenic organisms, including mycobacteria. Both biallelic LOF mutations (complete or partial deficiency) characterized by severe viral and bacterial infections during infancy and monoallelic LOF or GOF mutations (discussed further) have been described. AD STAT1 deficiency due to LOF mutations has been observed in different kindreds with milder forms of MSMD and incomplete penetrance (133). In a 3 generation Indian kindred with MSMD, the oldest patient with a p.G250A LOF mutation in STAT1 was 36 years at disease onset (141). Mutations can have a complete or partial LOF effect depending on their location and whether DNA binding capacity, STAT1 phosphorylation or both is affected (3). Moreover, for all mutations the effect on IFN-γ signaling is dominant negative in contrast to IFN-α and IFN-β signaling, explaining why most patients do not suffer from severe viral infections (3).

##### TYK2

TYK2 is a member of the JAK tyrosine kinase family and associates with multiple type I and II cytokine receptors. Recently, a genome wide study identified a common homozygous SNP in *TYK2* (p.P1104A) which is strongly enriched in European populations with a MAF of 4.2%, and is more prevalent in patients with tuberculosis (1%) compared to healthy individuals (0.2%) (134). Functionally this mutation was shown to carry a LOF effect by loss of TYK2 catalytic activity downstream of the IL23 receptor (135). Disease onset was highly variable (ranging from 1 to 40 years). Some patients received the BCG vaccine in early life without further complications and later developed pulmonary tuberculosis in adulthood, suggesting importance of pathogen virulence in addition to genetic susceptibility.

##### GATA2

*GATA2* encodes a transcriptional regulator of multilineage hematopoiesis. AD inherited LOF mutations can cause GATA2 deficiency syndrome, characterized by immunodeficiency (viral, bacterial, fungal, MSMD), hematopoietic disorders such as myelodysplastic syndrome (MDS) or acute myeloid leukemia (AML) and lymphedema, and a highly variable penetrance manifesting from infancy to adult age (142). A possible hypothesis for this variable penetrance is that the trigger of this syndrome might be evoked by somatic mutations in other genes such as *RUNX1, ETV6, CEBPA, ASXL1, SETBP1* and *STAG2*, as they commonly occur together (142). However, clear evidence about their influence on the pathogenesis is currently lacking. A series of 79 patients in France and Belgium was described recently and mycobacterial disease was observed in 8.1% of patients as the first presenting symptom, most of them after the age of 20 (136). In addition, hematological manifestations were often the first manifestation, with 69% presenting with MDS and 9% with AML. The median onset of symptoms in their GATA2 deficiency cohort was 18.6 years, ranging from 0 to 61 years of age.

#### 3.4.2 Herpes Simplex Encephalitis

##### TLR3

Toll like receptor 3 (TLR3) recognizes double stranded RNA (dsRNA), which is produced by most viruses including HSV type 1. In a cohort of 120 patients (both children and young adults), 6 pathogenic functionally validated *TLR3* variants were found in 6 patients (139). Three out of 6 patients had HSE episodes in adulthood and 1 of them had a first episode at the age of 24 years. LOF mutations can be inherited in a AD or recessive manner, and different mechanisms underly TLR3 deficiency (hypomorphic, haploinsufficiency or dominant negative effect). Not surprisingly the hypomorphic variant (p.R867Q) was found recessively in the patient with an adult-onset phenotype. The TLR3 expression was normal but functionally, the mutation led to an impaired signaling upon Poly(I:C) stimulation of primary fibroblasts and a TLR3-deficient cell line which was transfected with this mutant.

#### 3.4.3 Invasive Fungal Infections

##### CARD9

*CARD9* encodes an adaptor protein downstream of C-type lectin receptors that recognize fungal components. Mutations in CARD9 – presumed LOF – are associated with predisposition to mucocutaneous and invasive fungal disease (IFD). Functionally they can negatively impact cytokine production in response to fungal ligands, neutrophilic killing and Th17 immunity (137). A recent review on CARD9 deficiency evaluated 58 patients from 39 kindreds with disease causing recessive *CARD9* mutations (137). Fungi typically belonged to the phylum Ascomycota (including *Candida*, *Trychophyton*, *Aspergillus*) and most patients were affected by a single fungus Clinical penetrance was complete, although for patients with IFD most cases were adult-onset (median age 18 years, range 3.5-52 years). This is intriguing because in contrast to MSMD, colonization/exposure to fungi such as C. albicans begins very early in life (for C. albicans before the age of one in 50% of cases) (143).

#### 3.4.4 Chronic Mucocutaneous Candidiasis

##### STAT1

AD GOF *STAT1* mutations lead to defective Th1 and Th17 responses with a reduced production of IFN-γ, IL-17 and IL2, thereby leading to a phenotype of CMC, susceptibility to bacterial and viral infection and autoimmunity. The largest cohort till present studied 274 patients from 167 kindreds with STAT1 GOF mutations. Adult-onset was mostly seen in patients without CMC (n=6, range 4-61 year) whereas CMC often presented in early life (n=268, range birth-24 years) (138). Some patients might present first with mild auto-immune features such as autoimmune hypothyroidism, and then later in life during adulthood develop infectious complications such as CMC and severe viral infections with immunophenotypic abnormalities raising a suspicion for STAT1 GOF (144). Others may present in adulthood with IFD as the first manifestation or with multiple auto-immune or autoinflammatory features (such as Takayasu arteritis and inflammatory bowel disease) (145).

#### 3.4.5 Other Inborn Errors of Immunity Related to Leukocytes

##### IRF4

IRF4 is a transcription factor with essential functions in lymphocytes including development, antibody affinity maturation and roles in effector T cells. In a family affected by Whipple disease with a mean age of 55 years at onset, a private p.R98W mutation in *IRF4* was identified, proven to be LOF *in vitro* based on its decreased ability to bind DNA and to induce transcription of interferon stimulated response element motif containing promotors compared to WT IRF4 (140). IRF4 deficiency was identified as an AD cause of Whipple disease with (unexplained) incomplete penetrance.

### 3.5 Autoinflammatory Disorders

#### 3.5.1 Interferonopathies

##### CECR1

Deficiency of adenosine deaminase 2 (DADA2) was first described in 2014 in 9 patients with intermittent fever, systemic vasculopathy early-onset ischemic stroke (164), caused by recessive mutations in *CECR1* encoding ADA2. These mutations cause (near) complete absence of enzyme function resulting in elevated extracellular adenosine leading to dysregulated formation of neutrophilic extracellular traps, neutrophilic activation, polarization of macrophages from M2 to M1 subtype and increased proinflammatory cytokine production (165). Adult-onset cases have been documented in cohorts with idiopathic polyarteritis nodosa (160, 161) without prior manifestations although a thorough history should always be taken for possible features in infancy which retrospectively can be connected to DADA2 (**Table 6**) (166).

**Table 6:** Genes associated with adult-onset autoinflammatory diseases.

	Disease	Genetic defect	Inheritance	Functional defect	Phenotype (key features)	Reference
**AUTOINFLAMMATORY DISEASES**	VEXAS (vacuoles, E1 enzyme, X linked, autoinflammatory, somatic) syndrome	*UBA1*	Somatic X-linked , myeloid restricted	LoF, mutations affect translation initiation site and promote production of a hypomorphic UBA1c isoform leading to defective polyubiquitination	Fever, neutrophilic dermatosis and vasculitis, ear and nose chondritis, venous thrombosis, bone marrow myelodysplasia and vacuolization	(11)
	CAPS (cryopyrin associated periodic syndrome)	*NLRP3*	Germline dominant/somatic	GoF, mutations activate NLRP3 inflammasome assembly leading to excessive IL-1β production	Intermittent fever, neutrophilic urticaria, conjunctivitis, and arthralgia	(13, 146, 147)
	NLRP12 autoinflammatory disease	*NLRP12*	Germline dominant	Complex, LoF or GoF effect, reduced NF-κB inhibition potential or increased inflammasome activation	Intermittent fever, neutrophilic urticaria, conjunctivitis, and arthralgia	(148, 149)
	TRAPS (TNF receptor associated periodic syndrome	*TNFRSF1A*	Germline dominant (mostly low penetrance)/somatic	LoF, disturbs soluble receptor shedding, production of mutant TNF-R1 with intracellular sequestration and ER stress, upregulation of unfolded protein response, impaired autophagy	Intermittent fever, abdominal pain, myalgia, arthralgia, erythematous skin rash, periorbital edema, amyloidosis	(150–152)
	DITRA (deficiency of IL36 receptor antagonist)	*IL36N*	Germline recessive	LoF, loss of IL36 antagonist results in uncontrolled IL-36 induced inflammatory response in keratinocytes	Generalized pustular psoriasis	(153–156)
	FMF (Familial Mediterranean Fever)	*MEFV*	Germline dominant or recessive and somatic	LoF, pyrin has an anti-inflammatory role by inhibiting IL-1β production	Intermittent fever, peritonitis, skin rash, serositis, myalgia, arthritis and arthralgia	(157, 158)
				GoF, mutated pyrin forms a complex with apoptosis associated speck like protein to form its own inflammasome, inducing IL-1β production		
	Blau syndrome	*NOD2*	Somatic	GoF, NOD2 becomes constitutively active promoting the activation of NF-κB and proinflammatory cytokine production	Non caseating granuloma formation, dermatitis, uveitis	(159)
	DADA2 (deficiency of deaminase 2)	*CECR1*	Germline recessive	LoF, decreased ADA2 enzyme activity	Ischemic stroke, PAN vasculitis, livedoid rash, cytopenia, infectious susceptibility, lymphoproliferation	(160, 161)
	SAVI (Sting Associated Vascolupathy of Infancy)	*STING1*	Germline dominant	GoF, spontaneous activation of STING (dimerization or spontaneous trafficking from ER to Golgi) resulting in excessive type I IFN response	Digital ischemia, chilblain, interstitial lung disease and fibrosis, fever, failure to thrive	(162, 163)
	A20 haploinsufficiency	*TNFAIP3*	Germline dominant	LoF, reduced expression of A20 resulting in impaired deubiquitination (K63 chains) with excessive activation of NF- κB	Behçet like disease, systemic inflammation, intestinal symptoms	(10)

Additional clinical and immunological features can be found in (35).

##### STING1

SAVI is an acronym that stands for STING Associated Vasculopathy of Infancy, described in 2014 (162). It is an AD inherited disease caused by GOF mutations in *STING1*, causing an uncontrolled activation of the cyclic GMP-AMP synthase (cGAS)-STING cytosolic DNA sensing pathway resulting in excessive type I interferon production (162). Recessive inheritance of GOF mutations has recently been described (167). Clinical manifestations mainly consist of pulmonary (interstitial lung disease, fibrosis), systemic (fever, failure to thrive) or skin manifestations (chilblains, digital ischemia) (163). Although the acronym suggests an infancy onset, adult-onset vasculitis with renal manifestations has been reported in a patient (163). Genetic modifiers such as SNPs in *STING1* itself or other interferon related genes such as *IFIH1* could impact disease severity (163) and viral exposure in a *STING1* GOF mouse model determined the development of pulmonary fibrosis (23).

#### 3.5.2 Defects Affecting the Inflammasome

##### MEFV

Familial periodic fever syndrome is the most common monogenic autoinflammatory disorder. It is generally caused by homozygous or CH mutations in *MEFV* encoding the protein pyrin, although patients with heterozygous mutations are reported where no second hit was found (157). Pyrin interacts with inflammasome components and caspase 1 to induce the production of IL-1β. Whether mutations in *MEFV* act as GOF or LOF remains a matter of debate and evidence exists to support both hypotheses (168). Up till now 61 (likely) pathogenic mutations have been reported in the Infevers database linked to a phenotype of FMF and most of them validated *in vitro*. Adult-onset phenotype is commonly seen and differs from childhood onset FMF with regards to genetic and clinical aspects. Adult-onset FMF (onset ≥20 years is associated with a lower prevalence of highly penetrant mutations (e.g. homozygous M694V) and clinical symptoms such as fever, peritonitis, pleuritis, arthritis and erythema are less observed compared to early-onset FMF (169). A somatic, heterozygous myeloid restricted mutation (p.R652H) was claimed to be responsible for late onset FMF in a middle aged Ashkenazi Jewish woman with a prior diagnosis of JAK2 positive polycythemia vera (PV). At the time of PV diagnosis, the *MEFV* mutation was observed at a very low level by Sanger sequencing on PBMCs, but reanalysis 4 years later when inflammatory symptoms commenced, showed that the *MEFV* mutation reached a MAF of 46% in PBMCs. This mutation was only observed in co-segregation with the JAK2 mutant suggesting a JAK2 driven clonal expansion of *MEFV* mutant containing cells (158).

##### NLRP3

Cryopyrin associated periodic syndrome (CAPS) is a group of diseases related to a defect in the protein cryopyrin (NLRP3). NLRP3 is a key component of the inflammasome functioning as a pattern recognition receptor (PRR) binding pathogen associated molecular patters (PAMP) such as products released from damaged cells (uric acid, extracellular ATP). Upon binding to PAMPs, recruitment of the NLRP3 inflammasome and adapter protein apoptosis associated speck-like protein (ASC) is initiated activating caspase-1 which mediates the production of proinflammatory cytokines such as IL-1β (170). ASC has also been shown to have prionoid activities that propagate inflammation (170). GOF mutations in *NLRP3* result in abnormal activation of the inflammasome causing excessive, uncontrolled inflammatory responses. Clinically three phenotypes have been described; familial cold autoinflammatory syndrome (FCAS), Muckle Wells syndrome (MWS) and neonatal onset multisystem inflammatory disease (NOMID). Although initially described in neonates and infants, adult-onset patients have been reported. In one series the median age of onset was 13 (range 4-40) (171) with the oldest case reported having a disease onset at the age of 46 (172). In addition, somatic NLRP3 mosaicism has been identified in adult-onset cases (13, 146). Some of these patients were given the diagnosis of Schnitzler disease prior to genetic diagnosis (147). Clinical presentation includes intermittent febrile episodes, fatigue, headache, neutrophilic urticaria, conjunctivitis, and arthralgia (173).

##### NLRP12

*NLRP12* encodes the protein monarch-1 which mainly functions as a suppressor of the (non)-canonical NF-κB pathway. However, NLRP12 can also be involved in inflammasome signaling and drive caspase-1 activation resulting in proinflammatory cytokine release (174). Several sporadic cases or families with heterozygous *NLRP12* mutations, presenting with an autoinflammatory disease highly resembling CAPS, have been described (148, 149). The pathogenesis is complex and seems to depend on the type of the mutation. For example a described nonsense mutation p.R284* was shown to be less effective in suppressing NF-κB activity consistent with a LOF effect. Other missense mutations such as p.R294Q or p.R352C rather directly increase speck formation and caspase-1 signaling suggesting a GOF effect (174). A recent case series, describes adult-onset (range 18-54 years) in 27% (8/29) of studied patients, all harboring a pathogenic missense mutation (p.F402L or p.G448A) (148, 149).

#### 3.5.3 Non Inflammasome Related Conditions

##### CARD14

CARD14 functions as a scaffold protein, highly expressed in keratinocytes, that regulates NF-κB signaling. Pathogenic missense variants in *CARD14* with a GOF effect resulting in amplified NF-κB responses in keratinocytes can cause a spectrum of skin conditions such as pustular psoriasis, psoriasis vulgaris and familial pytiriasis rubra vulgaris without the presence of systemic symptoms. Incomplete penetrance and considerable disease severity variability and onset (neonatal-83 years) are seen within and between families (175, 176). The degree of NF- κB signaling activity induced by a mutant is probably a determinant for disease onset, as the most severe phenotype of early-onset generalized pustular psoriasis was seen in a patient with a *de novo* p.E138A variant which was demonstrated to have the highest NF-κB activity in *in vitro* overexpression experiments compared to other pathogenic *CARD14* mutations (176).

##### IL36RN

IL36 receptor antagonist deficiency is a genetic disorder associated with generalized pustular psoriasis. It was first reported in nine Tunisian families sharing a homozygous missense mutation in *IL36RN* (p.L27P), leading to a decreased expression and thereby unable to inhibit proinflammatory cytokine production by patient keratinocytes upon stimulation by IL-36. A total of 16 affected individuals of whom 4 developed disease in adulthood were reported (153). Later, other reports on adult- onset pustular psoriasis have been made in association with homozygous complete LOF variants in *IL36RN* (154–156). The variation in age of onset was therefore attributed to other modifying genes and/or environmental factors, since no partial gene function *in vitro* was retained.

##### NOD2

Blau syndrome is a rare AD autoinflammatory syndrome, characterized by non-caseating granulomatous arthritis, dermatitis and uveitis. It is caused by GOF mutations in NOD2, an intracellular PRR, resulting in a spontaneous activation of NOD2 with downstream activation of NF-κB responsive genes. The classic Blau syndrome was reported once in an adult patient (22 years at onset) (159). He carried gonosomal NOD2 mosaicism (p.R334G) and his both children had early-onset symptoms (11.8 and 30 months). Amplicon based deep sequencing of the NOD2 gene showed a MAF on PBMC of 12.9% in the father and 49-51.5% in the children suggesting a gene dosage or cellular compartment effect. One other patient with somatic mosaicism has been published with a later onset and milder phenotype although this was still with a presentation in infancy (177).

##### TNFAIP3

A20 haploinsufficiency is caused by germline heterozygous LOF mutations in *TNFAIP3*, encoding the deubiquitination enzyme A20. A20 is a critical regulatory unit of the canonical NF-κB pathway, by functioning as an inhibitor of key proinflammatory molecules. The causal link between heterozygous LOF variants in A20 an autoinflammatory disorder with Behçet-like manifestations (aphtous stomatitis, genital ulcers and intestinal symptoms) was described in 2016 (178). Adult-onset cases (oldest age of onset 20 years) have been sporadically reported in families with variable penetrance (10).

##### TNFRSF1A

TNF receptor associated periodic syndrome (TRAPS) is the second most frequent inherited AD autoinflammatory disease. TNF-R1 expressed on immune cells is activated by TNF-α, allowing the recruitment of several adaptor proteins leading to the formation of complex I which activates NF-κB and transcription of anti-apoptotic and proinflammatory genes. Up till now 103 mutations, most of them located in the extracellular part of the receptor, have been classified as (likely) pathogenic in patients with a compatible phenotype according to the Infevers database. Some of these mutations still remain to be validated by *in vitro* assays. TRAPS is more complex than other autoinflammatory condition regarding the pathogenesis, since there is not one predominant mechanism. Mutations can have different and multiple impacts on protein function, either by affecting the cleavage of the extracellular domain preventing the release of soluble receptors (‘shedding effect’) attenuating inflammatory response or by expressing a mutant TNF-R1 alongside the WT resulting in a dysregulated inflammatory response (179). Adult-onset phenotypes are associated with low penetrance variants such as p.R92Q or p.P46L (150) but also somatic mosaicism has been described in two cases of adult-onset TRAPS (151, 152). Clinical features may range from atypical images such as isolated recurrent pericarditis to more typical but adult-onset periodic fever syndromes with serositis, myalgia/arthralgia, erythematosus skin lesions, periorbital edema, and, in case of long-standing uncontrolled inflammation, amyloidosis (173).

##### UBA1

VEXAS (vacuoles, E1 enzyme, X linked, autoinflammatory, somatic) syndrome is a recently described autoinflammatory disorder that presents in adult males, typically age 45-80 years (11). The underlying genetic defect is caused by somatic mutations in *UBA1*, an E1 ubiquitin activating enzyme. In a normal situation, two UBA1 isoforms (UBA1a and UBA1b) are produced from two translation sites (M1 and M41). The reported mutations (p.M41V, p.M41L, p.M41T) disrupt the second translation site (M41) of UBA1, resulting in the production of a third isoform (UBA1c) from a third downstream translation site (M67) which is normally not expressed. UBA1c, compared to UBA1a and b, is catalytically impaired resulting in loss of ubiquitylation leading to proteotoxic stress and dysregulated autophagy. Remarkably these mutations are restricted to the myeloid lineage in the periphery. Lymphoid progenitors were shown to carry the mutation, but for an undefined reason, mutated lymphoid progenitors do not further mature and translocate into the peripheral blood. Clinical presentation manifests as fever, nose/ear chondritis, skin disease with vasculitis and neutrophilic infiltration, venous thrombosis and often hematologic abnormalities ranging from isolated macrocytosis to myelodysplastic syndrome on bone marrow biopsy. Vacuolization of myeloid cells was present in all patients.

### 3.6 Complement Deficiencies

The complement system is a highly conserved part of our innate immunity, organized in three enzymatic pathways: the classical (CP), alternative (AP) and lectin pathways. Briefly, activation of these pathways results in the cleavage of C3 by a C3 convertase, followed by formation of a C5 convertase which cleaves C5 and initiates the activation of the common terminal pathway where a membrane attack complex (MAC) is formed by complement proteins C5b-9 (185). This MAC is responsible for the lysis of target cells (eg. bacterial, human cancer cells) promoting host defense. Deficiencies in complement are associated with auto-immune diseases such as systemic lupus erythematosus (SLE), frequently seen in defects of the early components of the classical pathway (*C1Q, C1R/S, C2, C4)* or with susceptibility to meningococcal disease and/or atypical hemolytic uremic syndrome (aHUS) by defects in the terminal pathway components (*C5, C6, C7, C8A/B/G, C9)* or complement regulators (*CFP, CFH, CFD, CFI, CD46*). Complement deficiencies in general are rare (~5% of IEIs), and seldomly have a first presentation in adulthood (**Table 7**) (186). If it occurs, adults mostly present with meningococcal disease or aHUS and genetic defects are found in the terminal component pathway or its regulators (186). For the scope of this review, focusing on immunodeficiency, we focused on genetic defects in adults with infectious susceptibility rather than isolated aHUS (with in rare cases infectious susceptibility) since the latter proportion of patients will likely be seen by a nephrologist. For an overview on C1-esterase inhibitor, encoded by *SERPING1*, the reader is referred to Busse et al. (183).

**Table 7:** Genes associated with adult-onset complement deficiencies.

	Disease	Genetic defect	Inheritance	Functional defect	Phenotype (key features)	Reference
**COMPLEMENT DEFICIENCIES**	C5	*C5*	Germline recessive	LoF, absent C5 levels	Meningococcal disease	(180)
	C6	*C6*	Germline recessive	LoF, absent C6 levels	Meningococcal disease	(180)
	C7	*C7*	Germline recessive	LoF , absent C7 levels	Meningococcal disease	(180)
	C8	*C8*	Germline recessive	LoF, absent C8 levels	Meningococcal disease	(180)
	C9	*C9*	Germline recessive	LoF, absent C9 levels	Meningococcal disease	(181)
	Factor D deficiency	*CFD*	Germline recessive	LoF, absent Factor D	Meningococcal disease	(182)
	C1q	*SERPING1*	Germline dominant	LoF, dominant negative, low levels of C1q (HAE type I) or reduced function (HAE type II)	Hereditary angioedema	(183)
	MASP2 deficiency	*MASP2*	Germline dominant/recessive	LoF, decreased expression/secretion and abolished lectin pathway activation	Herpes simplex encephalitis in adults	(184)

Additional clinical and immunological features can be found in (35).

#### C5, 6, 7, 8, 9

Phenotypically, deficiencies in the components of the MAC complex, are very resembling, presenting with invasive meningococcal disease (186). A nationwide French study enrolled 41 adults (defined as > 15 years) with diverse complement deficiencies and an infectious episode (180). Mean age at diagnosis was 28 years (range 15-67 years), with the highest proportion in group 15-25 years (25%). Importantly, half of the cohort already reported a serious infectious event before diagnosis (unspecified time of delay), so an adult-onset criterium is not always met. Genetic analysis demonstrated that 83% of patients had a terminal pathway deficiency (mostly in C7 and C6, followed by C5 and 8) and in 80% of the cases meningitis was the main clinical symptom. C9 deficiency was not found in this French cohort, because this is almost exclusively seen in patients from Japanese descent (181).

#### CFD

Factor D, encoded by *CFD*, is a peptidase and a component of the AP. It binds and cleaves factor B to Ba and Bb to promote downstream activation. Deficiency in Factor D, caused by a homozygous p.S42* mutation, was diagnosed in a 23-year-old, previously healthy, woman who presented with meningococcal disease at first presentation (182).

#### MASP2

*MASP2* encodes mannose binding lectin associated serine protease which forms a multimeric complex with mannose binding lectin and subsequently cleaves components C4 and C2 leading to downstream activation of the complement cascade (187). MASP2 deficiency was first described in 2013 in a patient who presented at the age of 13 years with ulcerative colitis and later in life developed infectious susceptibility (pneumococcal infections), skin involvement (erythema multiforme), progressive lung fibrosis and positive auto-immune antibodies (188). Functional assays showed a non-functional lectin pathway in this patient, caused by a homozygous missense mutation (p.D120G) in *MASP2*, leading to an abolished expression. Recently, a case of two patients with adult-onset HSE (24 and 60 years) was reported (184). Both of them had a single heterozygous deleterious mutations (p.G634R and p.R203W). Both mutations led to an abnormal protein secretion, a lost ability of auto-activation (p.G634R) or reduced antiviral activity (p.G634R). Furthermore, the authors showed that rare *MASP2* variants are enriched among HSE patients compared to healthy controls and that mice deficient in mannose binding lectin (MBL) were more prone to HSE (with lower survival rates and higher viral loads) upon intranasal inoculation (184). Whether MASP2 deficiency is a monogenic cause of IEI or merely a contributing factor is still a matter of debate (189). Healthy individuals who are MASP2 deficient (and have homozygous p.D120G mutations) have been reported. It might be possible that interplay with environmental triggers is important before a phenotype can manifest, or that there is a large redundancy for MASP2 in human defenses (189).

## 4 Conclusion

Advances in next generation sequencing has significantly expanded the identification of novel genes involved in IEI over the years, not only in early-onset severe IEI such as SCID but also in milder forms such as antibody deficiencies, innate immune defects, immune dysregulation diseases, autoinflammatory diseases and complement deficiencies that can manifest in adulthood. The spectrum of age of onset during adulthood is highly variable for all forms of IEI, except for the diseases affecting humoral and cellular immunity which usually manifest in the first decades (**Figure 3**). The elucidation of molecular drivers of IEI has important consequences towards the management of these patients since it can rationalize targeted treatment (eg. JAK-inhibition in interferonopathies, TNF-α inhibitors in DADA2, CTLA4 agonists in CTLA4 haploinsufficiency, sirolimus in ALPS, leniolisib in APDS), bone marrow transplant in some cases, provide prognostic information and inform genetic counselling. Therefore, physicians encountering adult patients with recurrent (common or rare specific) infections, autoinflammatory disorders or lymphoproliferation should be aware of the occurrence of IEI in this population and, if confirmed, consider the possibility of a monogenic driven disease. These patients should be referred to a specialized tertiary center for further diagnostics using targeted gene panels or whole exome/genome sequencing. Mechanisms for late onset disease are not always well understood, but hypomorphic mutations allowing partial protein function, somatic mosaicism, environmental exposure and epigenetics are likely the main contributors.

## Author Contributions

RS initiated and supervised the study. FS took the lead in drafting the manuscript. FS and TC reviewed the literature. All authors provided critical feedback and helped shaping the manuscript.

## Funding

FS (11B5520N) is fellow of the Fonds Wetenschappelijk Onderzoek - Vlaanderen National Fund for Scientific Research (FWO). RS is FWO senior clinical investigator fellows (1805518N, respectively) and received funding from KU Leuven C1 (C12/16/024). RS and SV are members of the European Reference Network for Rare Immunodeficiency, Autoinflammatory and Autoimmune Diseases (Project ID No 739543). This work was supported by the VIB Grand Challenges Program.

## Conflict of Interest

The authors declare that the research was conducted in the absence of any commercial or financial relationships that could be construed as a potential conflict of interest.

## Publisher’s Note

All claims expressed in this article are solely those of the authors and do not necessarily represent those of their affiliated organizations, or those of the publisher, the editors and the reviewers. Any product that may be evaluated in this article, or claim that may be made by its manufacturer, is not guaranteed or endorsed by the publisher.
